# RNA interference mediated mortality in *Aedes albopictus*: a challenging journey toward species-specific vector control

**DOI:** 10.1186/s13071-025-07113-2

**Published:** 2025-11-14

**Authors:** Bodunrin Omokungbe, Alejandra Centurión, Sabrina Stiehler, Magnus Wolf, Pascal Geisler, Andreas Vilcinskas, Antje Steinbrink, Kornelia Hardes

**Affiliations:** 1 LOEWE Centre for Translational Biodiversity Genomics (LOEWE TBG), Senckenberganlage 25, 60325 Frankfurt am Main, Germany; 2 Institute for Insect Biotechnology, Justus-Liebig University, Heinrich-Buff-Ring 26–32, 35392 Giessen, Germany; 3 Fraunhofer Institute for Molecular Biology and Applied Ecology IME, Branch of Bioresources, Ohlebergsweg 12, 35392 Giessen, Germany; 4 Institute for Evolution and Biodiversity (IEB), University of Muenster, Huefferstrasse 1, 48149 Muenster, Germany; 5 Senckenberg Biodiversity and Climate Research Centre (BiK-F), Georg-Voigt-Strasse 14-16, 60325 Frankfurt am Main, Germany; 6 BMBF Junior Research Group in Infection Research “ASCRIBE”, Ohlebergsweg 12, 35392 Giessen, Germany

**Keywords:** RNAi, dsRNA, dsRNases, Transfection reagents, Gene silencing, Nucleases, Arbovirus, dsRNA stability, RNAi formulation

## Abstract

**Background:**

*Aedes albopictus* is a major vector of pathogens, including arboviruses, causing thousands of deaths annually. With no effective antiviral therapies and increasing concerns about the ecological impact of chemical insecticides, species-specific strategies, such as RNA interference (RNAi), are beneficial. Thus, identifying and validating target genes that induce mortality is essential. However, RNAi efficacy in *Ae. albopictus* is often inconsistent, owing to multiple factors including degradation by nucleases. Therefore, molecular identification and quantification of the underlying nucleases will provide a basis for improving RNAi efficacy.

**Methods:**

Target genes were selected from previous studies, identified in *Ae. albopictus*, and their corresponding long double-stranded RNAs (dsRNAs) were designed. Using U4.4. cells as a first model, cytotoxicity was assessed with the CellTiter-Glo assay and gene knockdown via RT-qPCR. Larval survival assays and RT-qPCR were then used to evaluate in vivo effects. Owing to the lack of significant larval mortality, dsRNA complex size was analyzed using dynamic light scattering and their oral uptake was visualized by fluorescence microscopy. Suspecting degradation, dsRNA stability was assessed by agarose gel electrophoresis following incubation with larval gut extracts. This prompted the identification, characterization, and validation of two putative dsRNases. Finally, transfection reagents (TRs) were tested for their ability to protect dsRNA from degradation.

**Results:**

Only one of the synthesized dsRNAs targeting the inhibitor of apoptosis (IAP) significantly reduced U4.4 cell viability to 65% (uncomplexed-dsRNA) and 13% (K4-complexed dsRNA). However, all tested dsRNAs achieved significant gene knockdown in the cell-based assay. None of the dsRNAs induced significant larval mortality, because dsRNA was rapidly degraded by larval gut extracts within 4 min. Although, gene knockdown was confirmed in the gut tissue. Each of  the two identified dsRNases contained a signal peptide, catalytic residues, and substrate- and Mg^2^⁺-binding sites, and were highly expressed in larval guts. Of the dsRNA, 65% remained intact at 15 min when complexed with K4, but declining to 13% by 24 h.

**Conclusions:**

All target genes were significantly silenced in cells, and IAP in larval gut tissue. Although TRs improved dsRNA stability in vitro, no significant larval mortality was observed, likely due to rapid gut degradation. Therefore, effective RNAi-based control of *Ae. albopictus* requires identifying gut-specific essential genes and improved delivery systems.

**Graphical Abstract:**

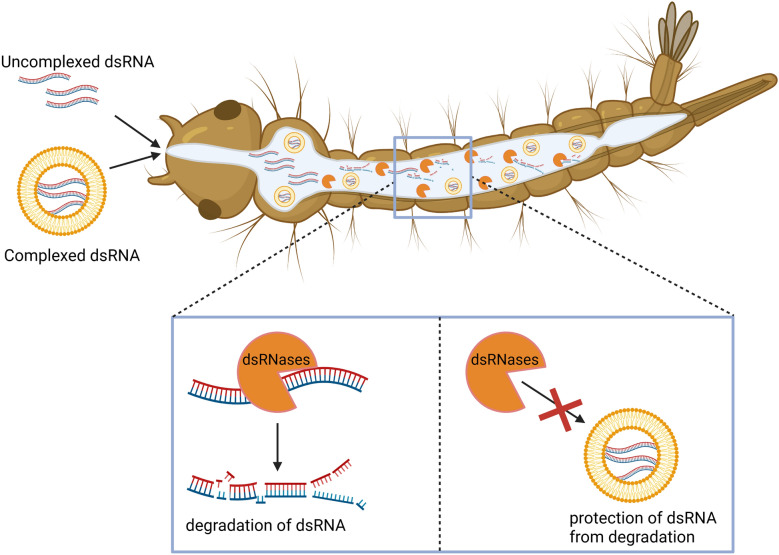

**Supplementary Information:**

The online version contains supplementary material available at 10.1186/s13071-025-07113-2.

## Background

Mosquitoes are known to transmit important pathogens that cause viral, bacterial, and parasitic diseases [1]. Among them, *Aedes albopictus* is of particular concern owing to its continuing worldwide expansion and its capacity to transmit up to 26 arthropod-borne viruses (arboviruses), including Zika virus, dengue virus, and Chikungunya virus [2, 3]. There are currently no specific treatments available for most diseases caused by the pathogens transmitted by *Ae. albopictus* [4]. Hence, reduction of the mosquito population relies heavily on the use of chemical insecticides [5]. However, these vector control methods face numerous challenges, including insecticide resistances and off-target effects [6]. For these reasons, the use of RNA interference (RNAi) as a species-specific vector control method by inducing targeted gene knockdown is of great interest [7].

RNAi is an evolutionarily conserved mechanism present in almost all eukaryotes, in which RNA molecules trigger sequence-specific knockdown of genes after transcription. There are three known RNAi pathways in mosquitoes: small interfering RNA (siRNA), micro-RNA, and piwi interacting RNA [8]. The siRNA pathway primarily functions as a defense mechanism against foreign nucleic acids and transposable elements [9, 10]. This pathway can be triggered by either siRNA or double-stranded RNA (dsRNA), with the latter being cleaved into ~21 nucleotide (nt) siRNAs by the enzyme Dicer-2. The siRNA is separated into two strands, with one strand loaded into the RNA-induced silencing complex. Together with the protein Argonaute-2, the complex binds to complementary mRNA, leading to its degradation [8, 11].

The utilization of long dsRNA as a successful biocontrol method for gene silencing in insects has been previously demonstrated [12]. RNAi has been used in several insects to target diverse genes, leading to a weakened immune system, causing mortality, or the inability to develop or reproduce [7, 13]. For example, silencing the beta-tubulin (β-tub) and inhibitor of apoptosis (IAP) genes led to high larval mortality in *Aedes aegypti* [14, 15]. Similarly, knockdown of genes such as Dre4, Ras opposite (ROP), and Nucampholin (NCM) led to high mortality in the red flour beetle *Tribolium castaneum* [16]. Despite the success of RNAi in various insects, it faces several challenges.

A key limiting factor is the inefficient and inconsistent uptake of dsRNA, since RNAi is only initiated when the dsRNA enters the cells. Thus, the delivery method largely determines its efficiency [17]. The most common methods of delivering dsRNA are microinjection, oral feeding, and soaking. For RNAi-based mosquito control, targeting the larval stages is most practical. For this, dsRNA can be added to breeding water for direct ingestion, a method simulated in the laboratory via soaking assays [18]. Another limiting factor includes the rapid degradation of dsRNA by gut nucleases [19]. The degradation is mostly mediated by the activity of dsRNA-degrading nucleases (dsRNases), which belong to the DNA/RNA nonspecific endonuclease superfamily and are predominately expressed in the gut to digest exogenous nucleic acid [20–22]. Although, knowledge of these limitations on the use of RNAi for mosquito control is not new, specific molecular parameters such as dsRNA degradation time and identification of specific dsRNases need to be defined to help overcome them. Additionally, the use of carrier systems has been investigated to better understand cellular uptake [23], as well as to improve RNAi efficacy by protecting dsRNA from degradation, e.g., in *Ae. aegypti* [14]. We therefore hypothesized that encapsulating dsRNA in carriers, such as liposomes, can enhance its stability and uptake in *Ae. albopictus* larvae, improving gene knockdown and RNAi-induced mortality.

This study aimed to induce larval mortality in *Ae. albopictus *via RNAi. We selected and identified homologs of β-tub, Dre4, IAP, ROP, and NCM genes, previously shown to cause high mortality in other insects [14–16]. Initial evaluation of dsRNAs targeting these genes, both complexed with liposome-based transfection reagents (TRs) and uncomplexed, was conducted in U4.4 cells using cell viability and gene knockdown assays. We then tested the dsRNAs in larvae through survival assays and gene knockdown analyses. Owing to a lack of significant mortality in larvae, we examined the particle size of the dsRNA complexes and visualized their oral uptake, to identify potential limiting factors. We also assessed dsRNA degradation by larval gut extract and subsequently identified, characterized, and confirmed the presence of dsRNase genes in *Ae. albopictus*, with high activity in the gut. Finally, we tested whether TRs were able to protect the dsRNA from gut degradation in vitro. These findings advance the study of RNAi as a potential biocontrol strategy against *Ae. albopictus* and related species, by providing for the first time, precise quantification of nuclease activity, together with molecular identification and characterization of the specific dsRNase genes underlying RNAi inefficacy in this mosquito.

## Methods

### Mosquito rearing

*Ae. albopictus* (Rimini strain) were obtained as dried eggs (provided by Dr. Hanano Yamada, Joint FAO/IAEA Centre, Vienna, Austria). Eggs were hatched using a vacuum pump system. Larvae were raised in plastic trays containing dechlorinated tap water and fed with pulverized Pleco tablets (Tetra, Melle, Germany). Emerged adults were supplied with 8% fructose solution ad libitum. Females received defibrinated sheep blood (Thermo Fisher Scientific, Frankfurt, Germany) via a Hemotek membrane feeder (Hemotek, Blackburn, UK). The colony was maintained at 28 ± 2 °C and 75 ± 5% humidity, and a 12:12 h light–dark cycle in a climate chamber (Regineering, Pollenfeld, Germany).

### Cell culture

*Ae. albopictus* cell lines C6/36 (provided by Prof. Dr. Stefanie Becker, University of Veterinary Medicine, Hannover, Germany) and U4.4 (obtained from Friedrich-Loeffler-Institute, Greifswald, Germany) were maintained at 28 °C in Leibovitz’s L-15 Medium GlutaMax, supplemented with 10% fetal bovine serum, 1% penicillin/streptomycin, 1% MEM nonessential amino acids, and 1% tryptose phosphate broth (all from Thermo Fisher Scientific).

### Candidate gene target selection and double-stranded RNA design

To identify RNAi target genes in *Ae. albopictus*, we searched literature for previously validated genes in other insects. Sequences were retrieved from VectorBase or the National Center for Biotechnology Information (NCBI). The complete coding sequence collection of *Ae. albopictus* was used to construct a local BLAST database using the makeblastdb tool from NCBI BLAST + suite. Each gene was queried via BLASTN 2.9.0 + , and the top five hits were selected for high sequence similarity and reliable ortholog identification. Only full-length mRNA sequences were retained. Genes known to induce high mortality were chosen: β-tub and IAP from *Ae. aegypti* [14, 15]; NCM, ROP, and Dre4 from *Tribolium castaneum* [16]. Suitable target regions were identified using the siRNA-Finder v21 (siFi21) [24]. Two dsRNA constructs per gene were designed using NCBI Primer-BLAST (400 and 500 bp), for regions identified by si-Fi21 as the main target. T7 promoter sequence was incorporated to the 5’ end of each primer for in vitro transcription. Primer details are provided in Table S1 (Supplementary Information).

### Double-stranded RNA preparation

For the synthesis of dsRNA targeting β-tub, Dre4, ROP, IAP, and NCM, total RNA from L4 *Ae. albopictus* larvae was extracted using the Monarch Total RNA Miniprep Kit (New England Biolabs, Frankfurt, Germany). Gene-specific primers with T7 promoter sequences were used for RT-qPCR with OneTaq One-Step RT-PCR Kit (New England Biolabs) on Applied Biosystems SimpliAmp thermal cycler (Thermo Fisher Scientific). The PCR products were purified using the NucleoSpin Gel and PCR Clean-up Kit (Macherey–Nagel, Düren, Germany), and used for in vitro transcription with the MEGAscript T7 Transcription Kit (Thermo Fisher Scientific). The dsRNAs were purified using lithium chloride precipitation, resuspended in nuclease-free water (Thermo Fisher Scientific), quantified by NanoDrop 2000 spectrophotometer (Thermo Fisher Scientific) with a dilution factor of 46.0, and stored at –80 °C. For mCherry dsRNA, a glycerol stock of *Escherichia coli* NEB 5-α (New England Biolabs) carrying pCMV-SFV6-2SG-mCherry (provided by Prof. Dr. Andres Merits, University of Tartu, Estonia and Prof. Dr. Andreas Pichlmair, Technical University of Munich, Germany) was cultured in lysogeny broth with 125 µg/mL kanamycin at 37 °C, 200 rpm overnight. GFP dsRNA was prepared similarly using *E. coli* harboring pGEM-T-Easy-GFP-125. Plasmid DNA from both strains was extracted using the NucleoSpin Plasmid DNA Kit (Macherey–Nagel). OneTaq Hot Start Quick-Load 2 × Master Mix (New England Biolabs) was used for PCR amplification of mCherry and GFP templates, followed by in vitro transcription as described above.

### Cytotoxic effects of RNAi in aedine cell lines

To assess RNAi-induced cytotoxic effects, two dsRNA constructs per gene were tested on U4.4 and C6/36 cells at ~50% confluency. C6/36 cells served as RNAi-deficient control [25–27]. Treatments were applied either uncomplexed or complexed with TRs, and then the plates were incubated at 28 °C. In a first analysis, uncomplexed dsRNA at 25 ng/µL per well was used. Concentrations were based on pilot experiments (data not shown). In the second analysis, dsRNA at 2 ng/µL either uncomplexed or complexed (dsRNA:TR) with K4 Transfection System (1:1, K4, Biontex, Munich, Germany), Metafectene Pro (1:07, Biontex), or CellFectin II (1:5, Thermo Fischer Scientific), were used based on our previous protocol [27]. All treatments were prepared in antibiotic-free supplemented L-15 medium. Controls included nuclease-free water, Grace’s insect medium, TRs alone, untreated cells, and ionomycin (100 µM in the assay, Thermo Fisher Scientific). At 6 h post-treatment (hpt), medium was replaced with fully supplemented L-15 medium. Cell viability was measured at 48 hpt using the CellTiter-Glo Luminescent Cell Viability Assay (Promega, Walldorf, Germany). Data were normalized to the untreated control.

### Larval bioassay for evaluating dsRNA-mediated mortality

Two larval bioassays were designed to assess RNAi-induced mortality. In the first, L1-L2 larvae were exposed for 4 h to uncomplexed or K4-complexed dsRNA (1:1) at 100 ng/µL in 75 µL nuclease free water inside 1.5 mL tubes. Larvae and solution were then transferred into 24-well plates (10 larvae/well, 3 replicates), and the volume was adjusted to 500 µL with tap water. Grounded larval food solution (25 µL) was provided daily, and mortality was recorded every 24 h. In the second approach, larvae were directly incubated in 24-well plates with 500 µL K4-complexed dsRNA at 20 or 50 ng/µL, and kept in the solution throughout the experiment. Feeding and monitoring followed the same procedure.

### Gene silencing analysis in U4.4 cells and larvae of *Aedes albopictus*

Prior to gene silencing analysis, primer efficiency was determined by standard curves (0.1–1000 ng, see Supplementary Information Sect.  3 for details). Gene knockdown was evaluated by RT-qPCR following dsRNA treatment. U4.4 cells were treated with 2 ng/µL K4-complexed dsRNAs, as described in the cytotoxicity assay. Total RNA was extracted at 48 hpt for the β-tub 1, Dre4 1, NCM 1, and ROP 1 dsRNAs, and at 24, 48, and 72 hpt for IAP 2 using the Monarch Total RNA Miniprep Kit. Larval RNA was extracted at 3 d post-treatment from whole bodies or dissected guts using the second larval bioassay approach described above. RNA quality was verified with a NanoDrop 2000. RT-qPCR was performed using the Luna Universal One-Step RT-qPCR Kit (New England Biolabs) on a QuantStudio 3 Real-Time PCR System (Applied Biosystems, Thermo Fisher Scientific). Details of reagents and cycling conditions are listed in Table S2. Actin was used as reference gene; mCherry dsRNA served as negative control. Relative expression was calculated via the 2^–ΔΔCt^ method.

### Dynamic light scattering analysis of formulated dsRNA

To assess whether dsRNA:TR complex size limited larval mortality, we analyzed them by dynamic light scattering (DLS) at concentrations of 10, 20, or 50 ng/µL. See Supplementary Information Sect.  4 for detailed description.

### Fluorescence uptake analysis of dsRNA in larval stage of *Aedes albopictus*

To assess dsRNA uptake, mCherry or IAP 2 dsRNA was labeled with Cy3 fluorophore using the Silencer siRNA Labeling Kit (Thermo Fisher Scientific). Labeled dsRNA, either uncomplexed or complexed with K4 (1:1) was incubated with larvae in breeding water for 1 h. Larvae were then rinsed and transferred to fresh wells containing clean tap water. Larvae incubated in tap water served as negative control. Fluorescence was examined at 1, 3, 6, 24, and 48 hpt using a M165 FC fluorescence microscope with EL6000 light source. Images were captured with a DFC450 C camera and processed using LAS-X v4.13 (All from Leica Microsystems, Wetzlar, Germany).

### Ex vivo degradation of dsRNA with gut extract of *Aedes albopictus*

In total, 50-pooled L4 larval guts were dissected in 500 µL nuclease-free water on ice and homogenized with cold ceramic beads using a TissueLyser II (Qiagen, Hilden, Germany) for 2 min at 30 Hz. After two centrifugation steps at 4 °C, supernatants were collected. To assess degradation, 10 µL of gut extract (≈1 gut) was incubated with 1 µg of mCherry dsRNA for 1–8 min. Reactions were stopped with 5 µL of 50 mM EDTA [21]. For the EDTA-containing gut extract experiment, EDTA was added to the gut extract prior to dsRNA incubation for 10 min. Samples were run on 2% agarose gels (110 V, 150 mA, 40 min) with the Mass Ruler loading dye (Thermo Fisher Scientific). A Gel Doc™ XR + (BioRad Laboratories, Munich, Germany) was used to visualize the gel bands. The ImageLab v.5.2.1 (BioRad Laboratories) was used to capture images and analyze the relative band intensity. Data were normalized to the controls.

### Identification and characterization of *Aedes albopictus* dsRNase proteins

*Ae. albopictus* dsRNases were identified by BLASTp search against its genome (NCBI RefSeq: GCF_035046485.1), using *Ae. aegypti* dsRNases as queries [28]. Protein features were predicted using the NCBI’s conserved domain search (https://www.ncbi.nlm.nih.gov/Structure/cdd/wrpsb.cgi) and the InterProScan v99.0 [29]. The dsRNase proteins from 14 dipterans were retrieved and aligned by the ClusterW in MEGA v11. Phylogenetic analysis was performed using the Maximum Likelihood method with Poisson correction model and 1000 bootstrap replicates for the confidence value (%) of each branch.

### Expression profile of *Aedes albopictus* dsRNase genes

For developmental gene expression analysis of dsRNases, total RNA was extracted from all larval stages (L1–L4), dissected L4 guts, and the remaining body tissue using the Monarch Total RNA Miniprep Kit. Sample sizes included 50 L1, 30 L2, 15 L3, and 10 L4 larvae, and 30 L4 larvae for gut dissections. RT-qPCR was performed using primers for Aal-dsRNase1 and Aal-dsRNase2 with the Luna Universal One-Step RT-qPCR Kit. Reference genes included actin and protein phosphatase-2A. Data were analyzed using the extended-ΔCt method [30]. Primer sequences are in Table S1 (Supplementary Information).

To confirm specificity, Aal-dsRNase1 and Aal-dsRNase2 were amplified via RT-PCR using the OneTaq One-Step RT-PCR Kit. Amplicons were sequenced by LGC Genomics (Berlin, Germany), and aligned with the corresponding reference sequences (see Fig. S5).

### Protection of dsRNA from degradation using commercially available transfection reagents

To assess dsRNA protection, mCherry dsRNA was complexed with K4 (1:1), Metafectene Pro (1:0.7), Metafectene SI + (Biontex; 1:1.5), or Lipofectamine 2000 (Invitrogen, Thermo Fisher Scientific; 1:3), then incubated with L4 gut extract for 10 min. For K4, additional time points were tested at 0.25–24 h. Uncomplexed dsRNA served as negative control and the addition of EDTA to gut extract prior to incubation served as controls for complexed-dsRNA samples. To decomplex samples for agarose gel analysis, gel loading dye purple (6X) containing SDS (New England Biolabs) was added before loading.

### Statistical analysis and visualization

The graphical abstract was created with BioRender and data were analyzed using GraphPad Prism. One-way ANOVA with Dunnett’s or Šidák’s multiple comparison tests was used for gene knockdown analysis in cells and larvae, respectively. Larval survival was analyzed by Kaplan–Meier with Log-rank (Mantel–Cox) test. The expression of dsRNase and dsRNA protection assays were analyzed via one-way ANOVA.

## Results

### Selection of target genes for dsRNA synthesis

Target genes were selected based on previous studies in other insects reporting high RNAi-induced mortality. Homologous genes in *Ae. albopictus* with ≥ 70% sequence identity were identified using NCBI tools. Each sequence was screened with the si-Fi21 software [24] against a custom local database of the *Ae. albopictus* genome to ensure specificity. Two dsRNAs targeting high-efficiency regions of each gene were designed (Table 1). For synthesis, primers with T7-promoter sequences were used to amplify templates via RT-PCR, followed by in vitro transcription and purification.

**Table 1:** List of target genes and dsRNA candidates along with their efficient siRNA hits and query accession numbers

Gene	Abbreviation of dsRNA candidate	Efficient siRNA hits (si-Fi21)	Insect	Accession number	Citation
β-tubulin (β-tub)	β-tub 1	237	*Aedes aegypti*	XM_001655975	[15]
	β-tub 2	240			
Inhibitor of apoptosis (IAP)	IAP 1	195	*Aedes aegypti*	DQ993355.1	[14]
	IAP 2	202			
Dre4	Dre4 1	197	*Tribolium castaneum*	XM_967384.1	[16]
	Dre4 2	214			
Nucampholin (NCM)	NCM 1	218	*Tribolium castaneum*	XM_001811253.1	[16]
	NCM 2	181			
Ras opposite (ROP)	ROP 1	239	*Tribolium castaneum*	NM_001170684.1	[16]
	ROP 2	226			

### RNAi-induced cytotoxicity in aedine cell lines

**Fig. 1:**
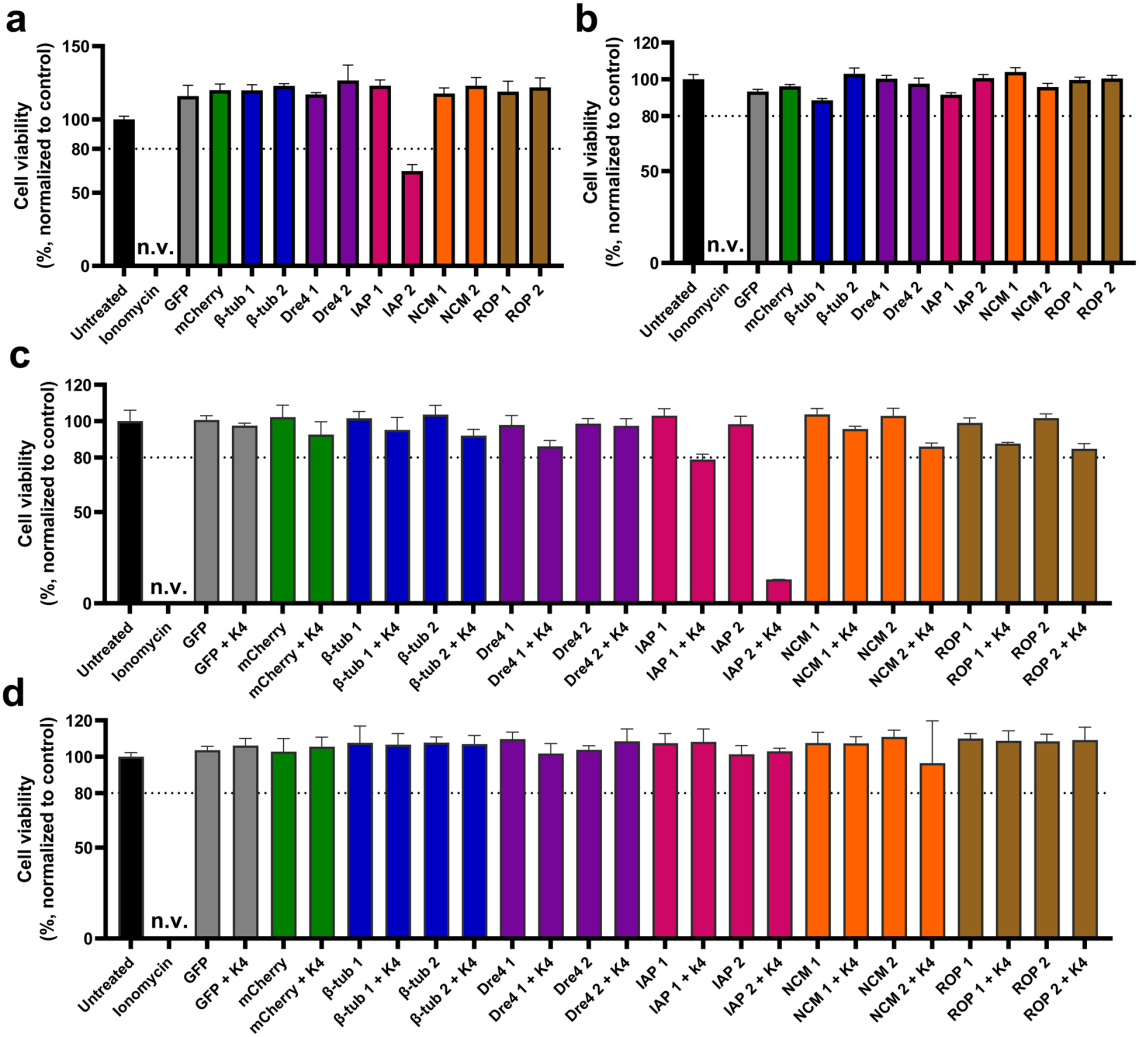
Cytotoxic effects of dsRNAs on aedine cell lines. Cells at ~50% confluency were treated with dsRNAs. **a** U4.4 and **b** C6/36 cells treated with uncomplexed dsRNAs at 25 ng/µL. **c** U4.4 and **d** C6/36 cells treated with dsRNAs complexed with K4 Transfection System at 2 ng/µL (1:1). Cell viability was assessed at 48 h post-treatment using the CellTiter-Glo assay. Data (*n* = 4) were normalized to untreated controls (treatment/control × 100). Bars show mean viability; error bars represent coefficient of variation (%). The dotted line indicates the 80% toxicity threshold. *n.v.* near-zero viability

To assess the cytotoxic effects of the synthesized dsRNAs, U4.4 cells were treated with 25 ng/µL of uncomplexed dsRNA targeting β-tub, Dre4, IAP, ROP, and NCM genes. At 48 hpt, cell viability was assessed using the CellTiter-Glo assay and only IAP 2 dsRNA significantly reduced cell viability in U4.4 cells to 65%, while the other dsRNAs had no cytotoxic effect (Fig. 1a). To confirm RNAi specificity, we used RNAi-deficient C6/36 cells as a control, where the dsRNAs had no cytotoxic effect (Fig. 1b).

We proceeded to transfect U4.4 cells with 2 ng/µL dsRNAs complexed with K4, CellFectin II, or Metafectene Pro. Only IAP 2 dsRNA reduced cell viability to 13% with K4 (Fig. 1c), 34% with CellFectin II, and 40% with Metafectene Pro (Fig. S1, Supplementary Information). Other dsRNAs showed no effect. Similarly, transfection of complexed dsRNAs into C6/36 cells showed no significant cytotoxicity (Fig. 1d).

### Gene knockdown analysis of complexed dsRNA in U4.4 cells

**Fig. 2:**
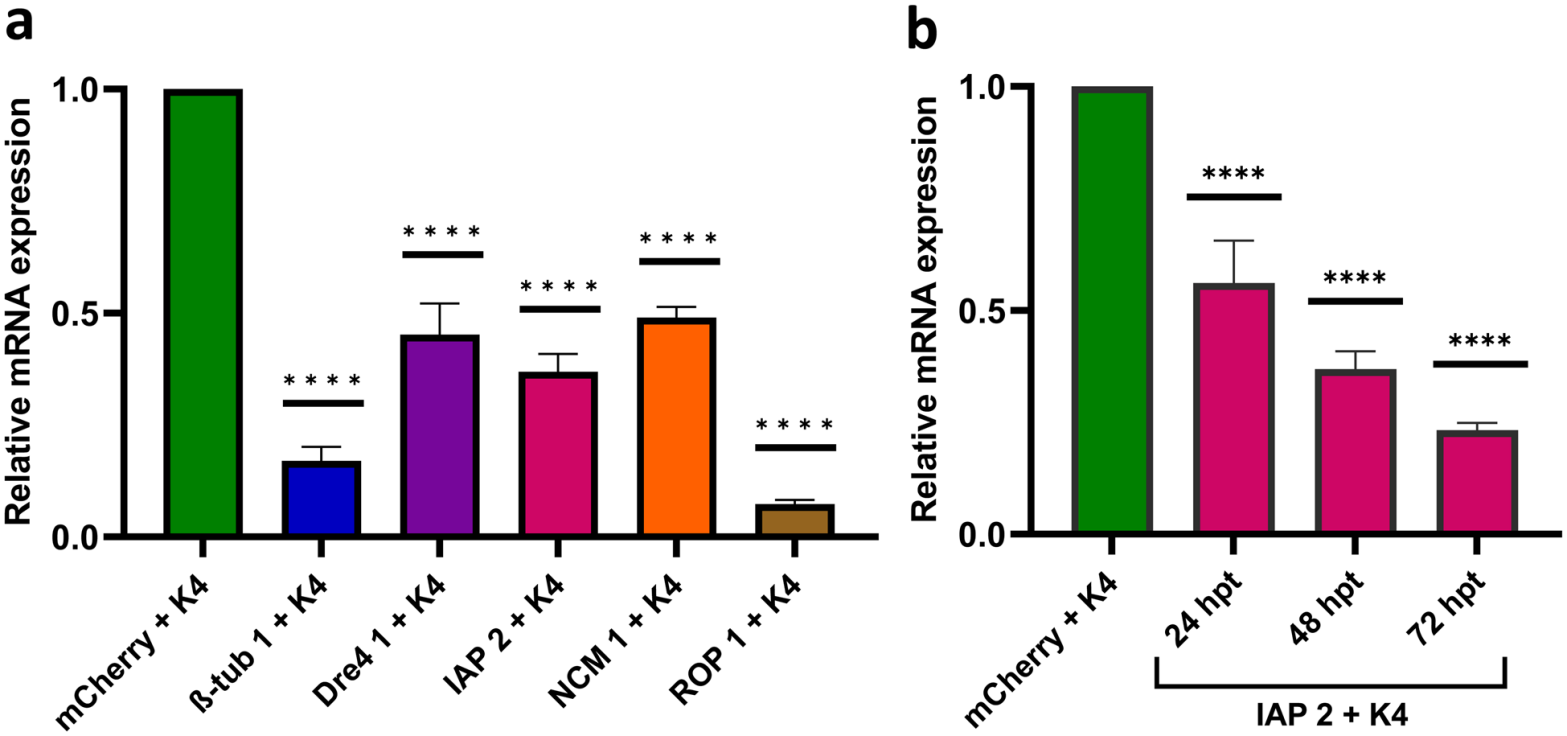
Gene knockdown analysis in U4.4 cells following treatment with complexed dsRNAs using the K4 Transfection System (1:1). **a** Gene knockdown analysis of selected dsRNAs at 48 h post-treatment (hpt). **b** Time-course knockdown analysis of the IAP 2 dsRNA at 24, 48, and 72 hpt. Total RNA was extracted at each time point and used for RT-qPCR. Data were analyzed using the 2^−ΔΔCt^ method. Gene expression levels are shown relative to the nonspecific control mCherry dsRNA (*n* = 3), with actin used as reference gene. Error bars represent standard deviations. The asterisk (****) represent a significant difference (*p* < 0.0001) to the control via one way ANOVA and Dunnett’s multiple comparison test

To further validate whether dsRNA-induced cytotoxicity was RNAi-specific, we selected one dsRNA variant of each gene for knockdown analysis via RT-qPCR. The mCherry dsRNA was included as a nonspecific negative control and actin as a reference gene for normalization. The dsRNAs were transfected into the cells using K4 following the standard protocol. At 48 hpt, all target genes showed reduced mRNA levels, with ROP 1 dsRNA achieving the highest knockdown of 93%, while NCM 1 dsRNA had only 51% knockdown (Fig. 2a). A time-course with IAP 2 dsRNA showed increasing knockdown over time, with 44%, 63%, and 77% for 24, 48, and 72 hpt, respectively (Fig. 2b).

### Larval mortality following dsRNA exposure in *Aedes albopictus*

**Fig. 3:**
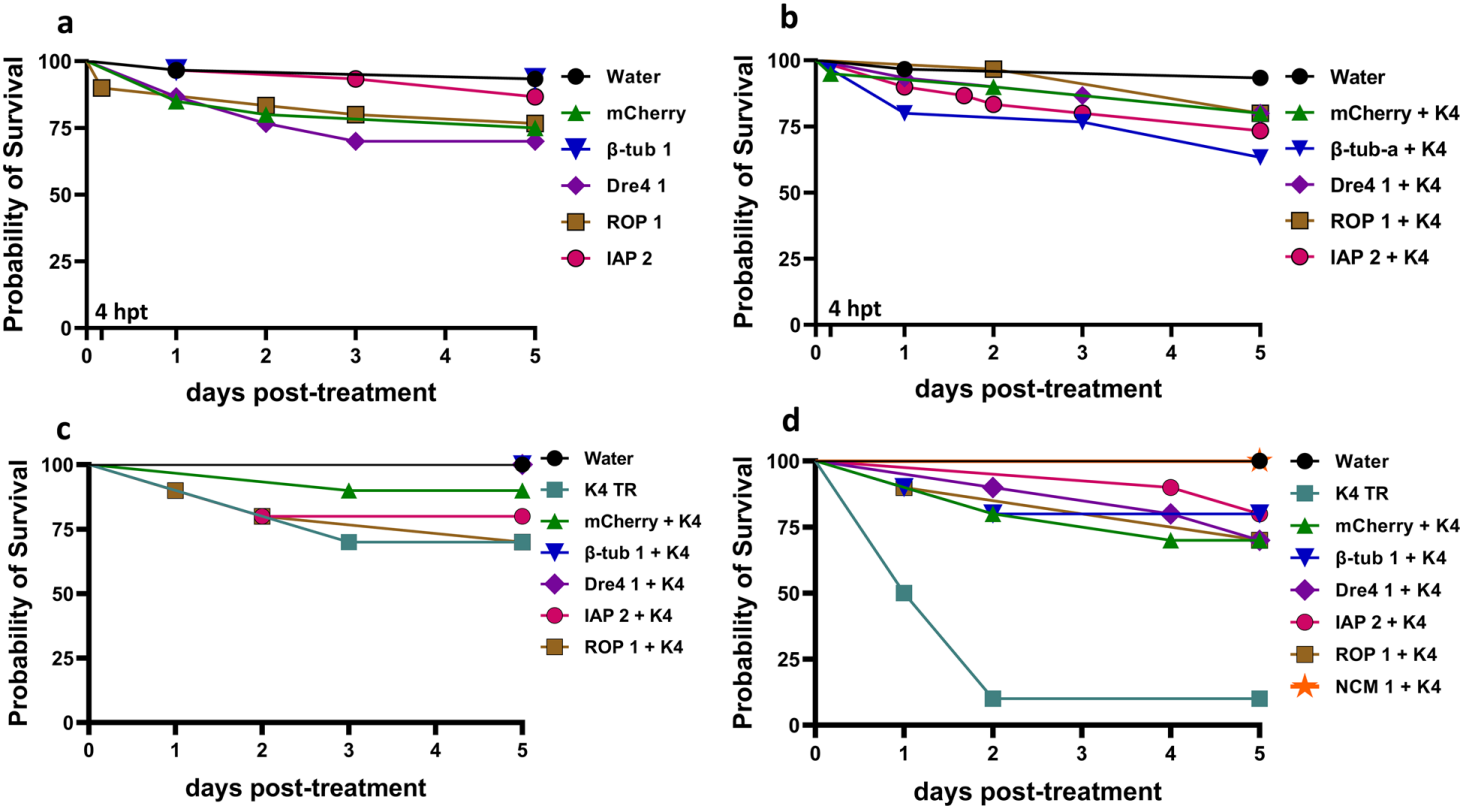
RNAi-induced mortality in *Aedes albopictus* L1–L2 larvae following dsRNA treatment. In method one, larvae were incubated in **a** uncomplexed or **b** K4-complexed dsRNA (1:1), at 100 ng/µL for 4 h, then transferred to 24-well plates with a final volume of 500 µL. In method two, larvae remained in K4-complexed dsRNA solutions at **c** 20 or **d** 50 ng/µL for the entire experiment. Larval mortality was recorded daily. Survival probabilities were estimated using the Kaplan–Meier method and compared with the log-rank (Mantel-cox) test. Data are shown as percentages

To evaluate dsRNA-induced mortality in vivo, L1-L2 larvae were exposed to dsRNAs targeting β-tub, Dre4, IAP, ROP, or NCM genes, with or without complexation with K4. While TRs are not commonly used in vivo, we employed them in this study as a proof-of-concept to assess whether complexation enhances RNAi efficacy, given that uncomplexed dsRNA rarely achieves significant mortality in this species. Tap water and mCherry dsRNA served as negative controls. Two exposure methods were used: In the first method, larvae were pre-incubated in 75 µL of dsRNA for 4 h (*n* = 30), then transferred into 24-well plates with the final volume adjusted to 500 µL. In the second method, larvae were directly exposed to dsRNA in 24-well plates (*n* = 10) for the entire duration of the experiment. Mortality was monitored daily in both cases. No significant mortality was observed with 100 ng/µL dsRNA in the first method (Fig. 3a and b), or 20 and 50 ng/µL in the second (Fig. 3c and d, respectively). However, in the second method, K4 alone showed toxicity at 50 ng/µL (Fig. 3d).

### Gene knockdown analysis of complexed dsRNA in larvae of *Ae. albopictus*

**Fig. 4:**
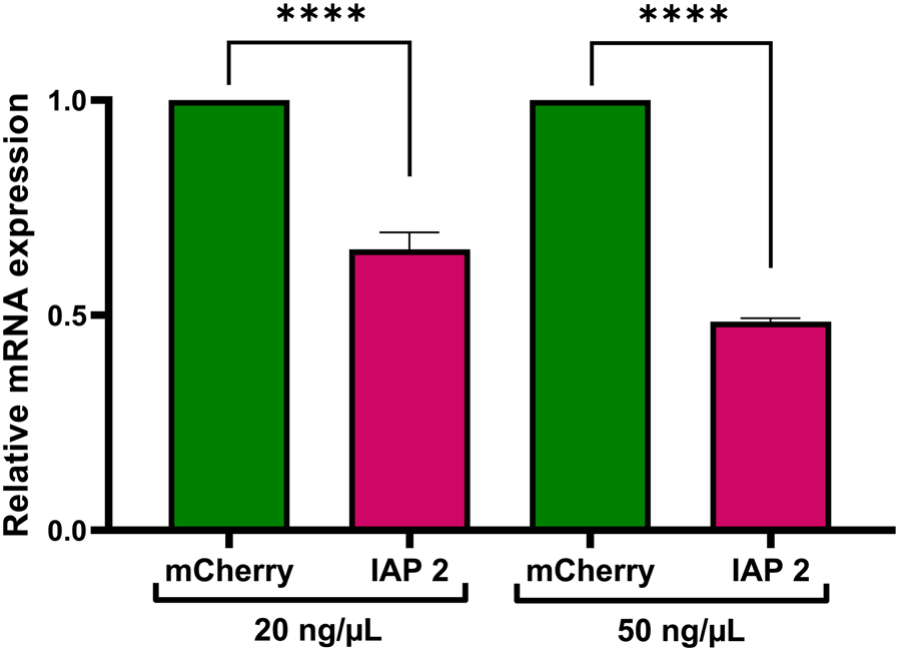
Gene knockdown in larval guts following treatment with complexed IAP 2 dsRNA using the K4 Transfection System (1:1). Larvae were treated using the second approach, and guts were dissected at 3 days post-treatment for RNA extraction and RT-qPCR analysis. Actin was used as the reference gene, and relative expression was calculated using the 2^−ΔΔCt^ method, normalized to the nonspecific mCherry dsRNA control (*n* = 3). Error bars represent standard deviation. Asterisks (****) indicate a significant difference (*p* < 0.0001) by one-way ANOVA and Šidák’s multiple comparison test

To assess the gene silencing despite the lack of significant mortality, IAP 2 dsRNA was selected for RT-qPCR, with mCherry dsRNA as the control and actin as the reference gene for normalization. Larvae were treated with K4-complexed IAP 2 dsRNA (1:1) following the protocol of the second bioassay method. At 3 days post-treatment dissected gut or whole larvae were used for RNA extraction. RT-qPCR revealed a reduction in IAP mRNA levels in the gut, with knockdown of 34.7% and 51.5% at 20 and 50 ng/µL, respectively (Fig. 4). However, no significant knockdown was detected when whole larvae were analyzed (Fig S3, Supplementary Information).

### Particle size determination and uptake dsRNA in larvae

Given the lack of RNAi-induce mortality, we investigated whether the dsRNA:TR complex size limited uptake. To determine the particle size of complexed mCherry dsRNA with K4, Metafectene Pro, or CellFectin II, we performed a DLS analysis using the side scattering method. The complex sizes formed at 10 ng/µL were similar, ranging between 150.6 and 165.8 nm (Supplementary Information, Table S3). The Polydispersity Index (PI) values were 0.10, 0.14, and 0.18 for K4, Metafectene Pro, and CellFectin II, respectively. In contrast, K4 complexes at 20 and 50 ng/µL were much larger (2461.7 and 5192.2 nm) with higher PI values (0.51 and 0.54).

**Fig. 5:**
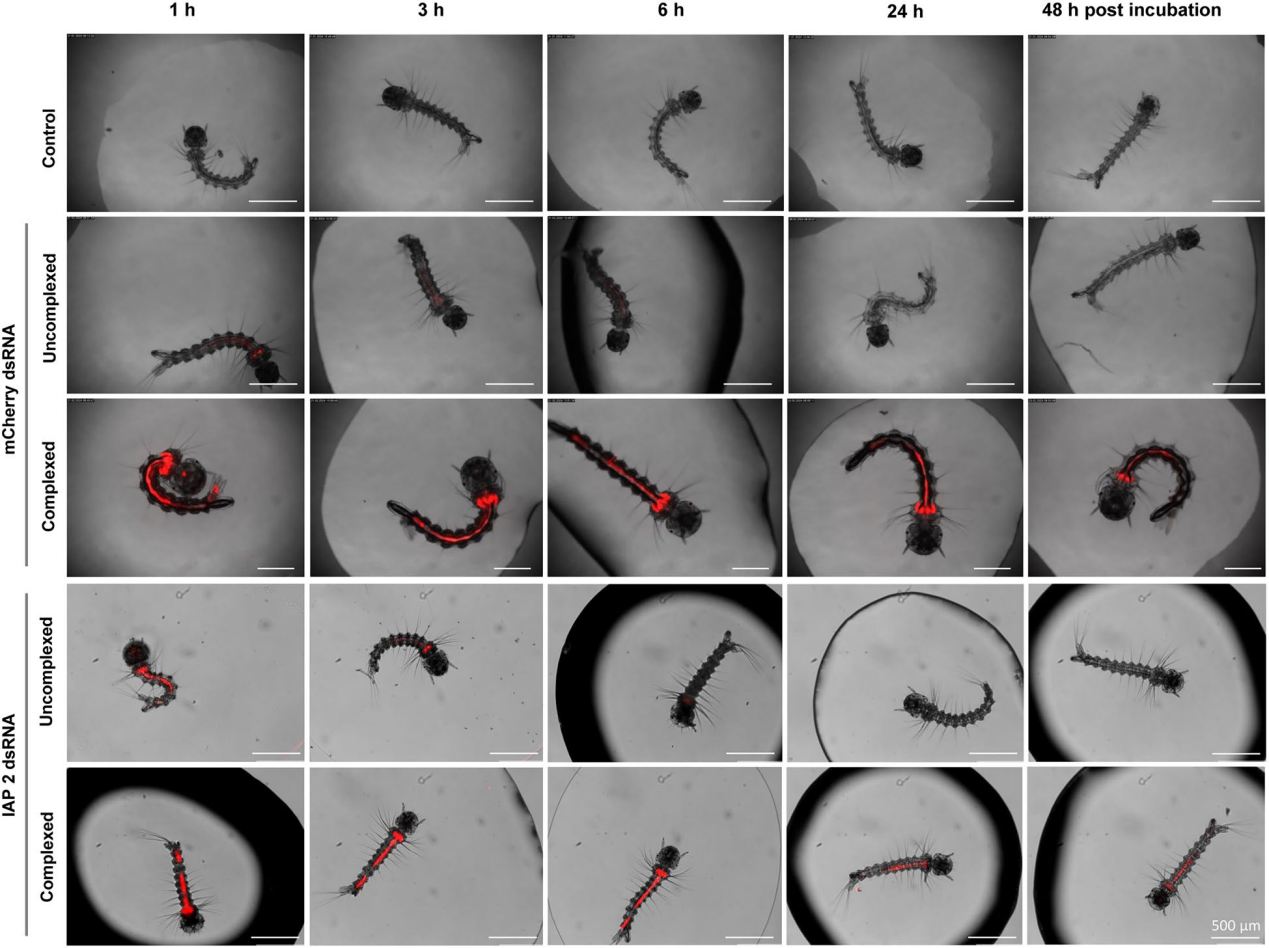
Uptake analysis of dsRNA in the gut of *Aedes albopictus* larvae. Larvae were exposed to Cy3-labeled dsRNA, either uncomplexed or complexed with K4 Transfection System at 200 ng/µL for 1 h. Following incubation, the larvae were transferred to clean breeding water. Fluorescence signals from the dsRNA were monitored at multiple time points. Scalebar = 500 µm

To assess the oral uptake, larvae were incubated for 1 h in uncomplexed or K4-complexed Cy3-labeled mCherry or IAP 2 dsRNA at 200 ng/µL. Larvae were rinsed and placed in clean water. Both forms were taken up, with more pronounced fluorescence signal observed in those incubated with complexed dsRNA (Fig. 5). Uncomplexed dsRNA signal was not detectable at 24 and 48 h post-incubation, whereas complexed dsRNA remained noticeable.

### Ex vivo degradation of dsRNA using *Aedes albopictus* gut extract

**Fig. 6:**
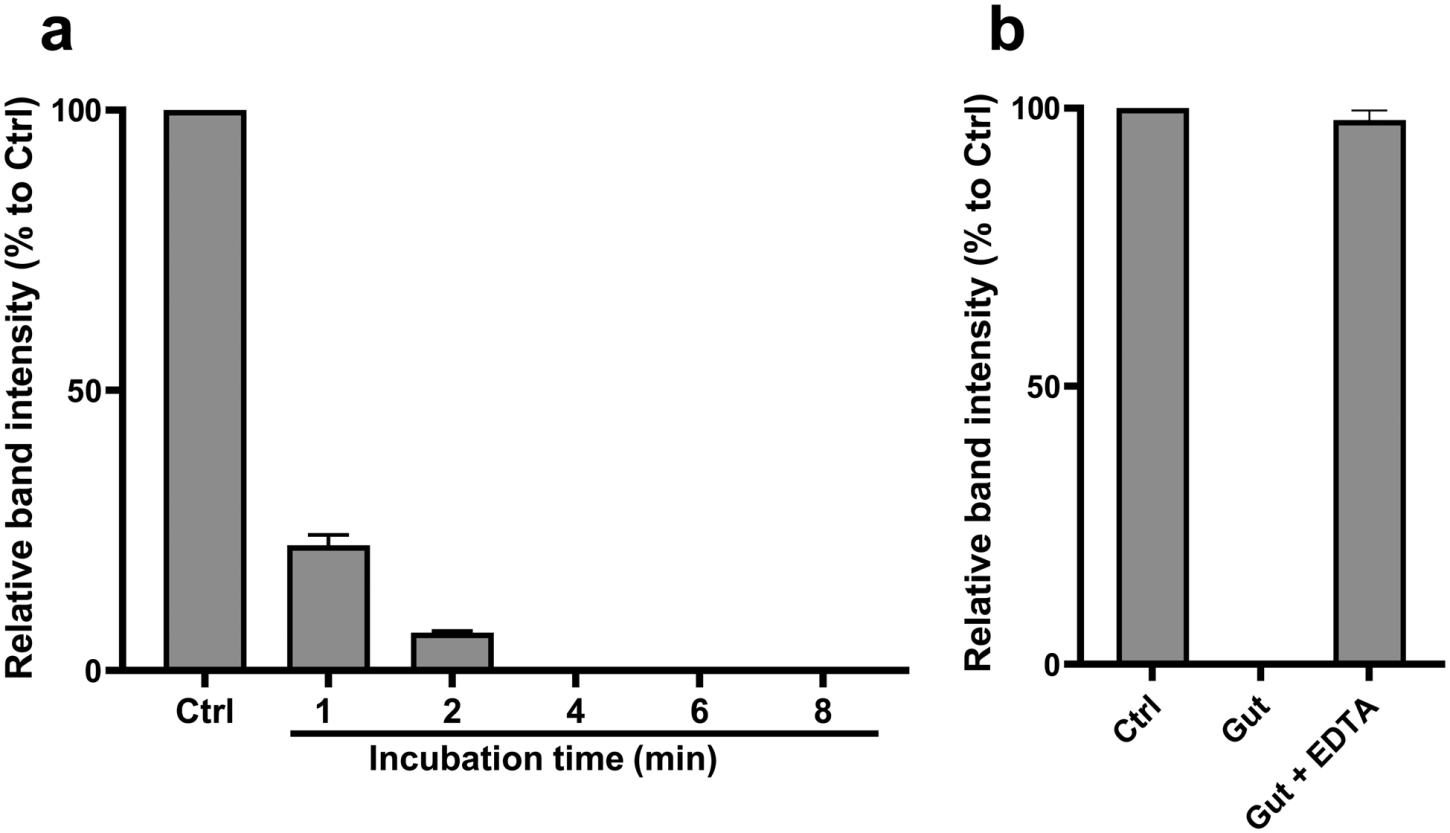
Ex vivo degradation of dsRNA using gut extract of *Aedes albopictus* L4 larvae. Gut extract was used to incubate mCherry dsRNA. In **a**, the dsRNA was incubated with the gut extract for 1–8 min. In **b**, the dsRNA was incubated for 10 min with either gut extract alone or mixture of gut extract and EDTA. All samples were resolved on agarose gel electrophoresis immediately after incubation and the relative band intensity was determined and normalized to the control (Ctrl). The data shown are the normalized mean (%) of three independent experiments (*n* = 3) and the error bar represents the standard deviation

To investigate whether gut nucleases contributed to the lack of RNAi-induced mortality, we tested dsRNA stability by incubating 1 µg of mCherry dsRNA with L4 larval gut extract. Degradation was assessed by analyzing relative band intensity in agarose gel electrophoresis. The ex vivo analysis revealed rapid degradation of dsRNA, with complete degradation observed within 4 min (Fig. 6a). Meanwhile, degradation was completely inhibited when EDTA was added to the gut extract prior to incubation (Fig. 6b). The agarose gel images can be found in Fig. S4 (Supplementary Information).

### Identification, characterization, and expression profile of *Aedes albopictus* dsRNases

**Fig. 7:**
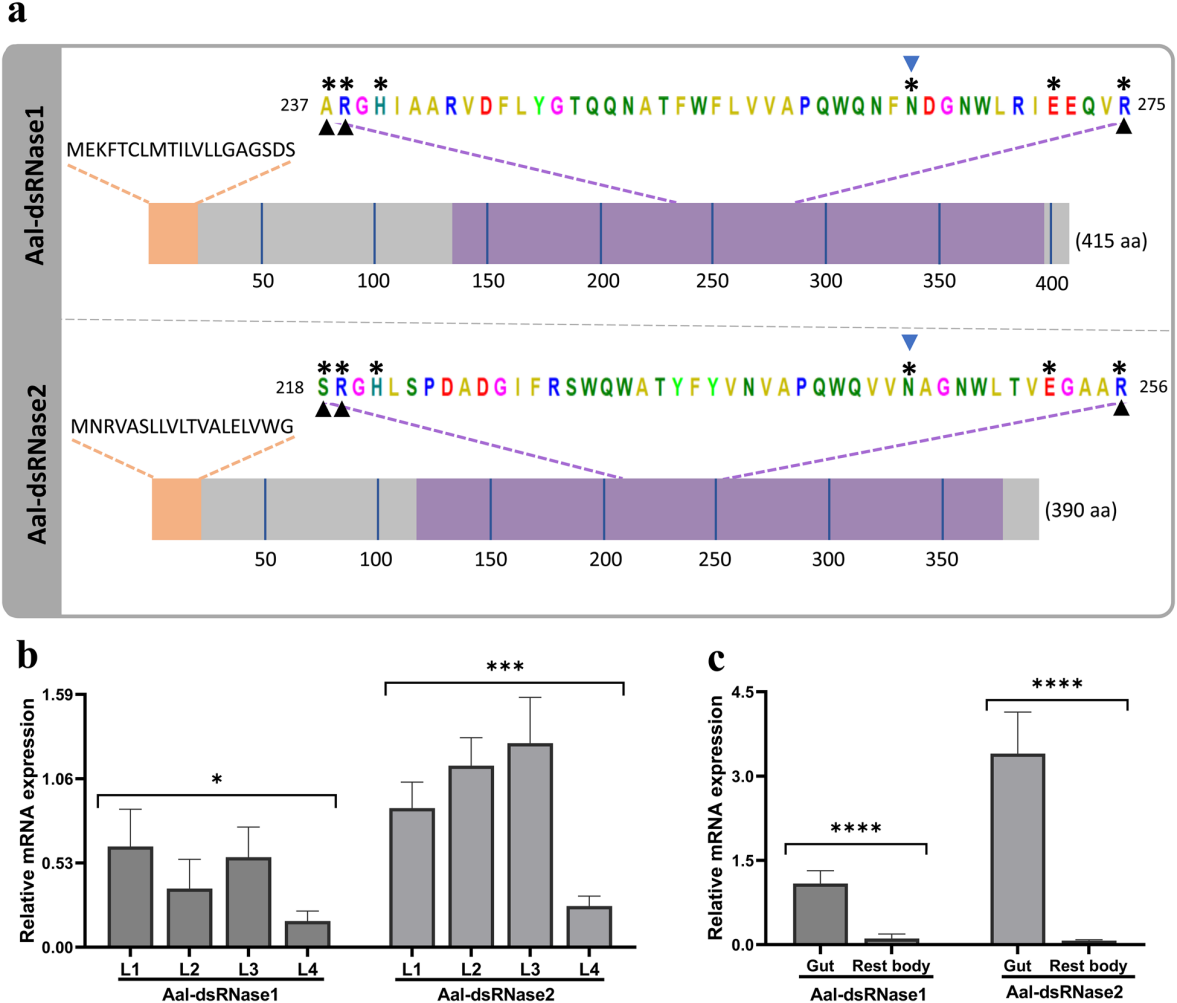
Characterization of *Aedes albopictus* dsRNase proteins and their gene expression profile. **a** Schematic illustration of the two retrieved dsRNase proteins, with their conserved domains and total length (in brackets). The sequences of the signal peptides are specified. The active site (*), substrate binding site (▲), and Mg^2+^ binding site () are also highlighted. The grey color indicates the noncytoplasmic domain, orange color shows the signal peptide region, while the purple color highlights the DNA/RNA nonspecific endonuclease domain. **b**, **c** Expression profiles of *Ae. albopictus* dsRNases. The expression of Aal-dsRNase1 and Aal-dsRNase2 was analyzed using RT-qPCR in **b** L1–L4 larval stages and in **c** dissected gut and rest body of L4 larvae. The relative mRNA level was calculated via the extended ∆CT method (e-∆CT) using actin and protein phosphatase 2A as the reference genes for normalization, and the values are expressed as 2^−∆CT^. The data shown are mean of three biological replicates (*n* = 3) and the error bars representing standard deviation. Asterisks (*, ***, and ****) indicate a significant difference (*p* < 0.05, *p* < 0.001, and *p* < 0.0001) by one-way ANOVA

**Fig. 8:**
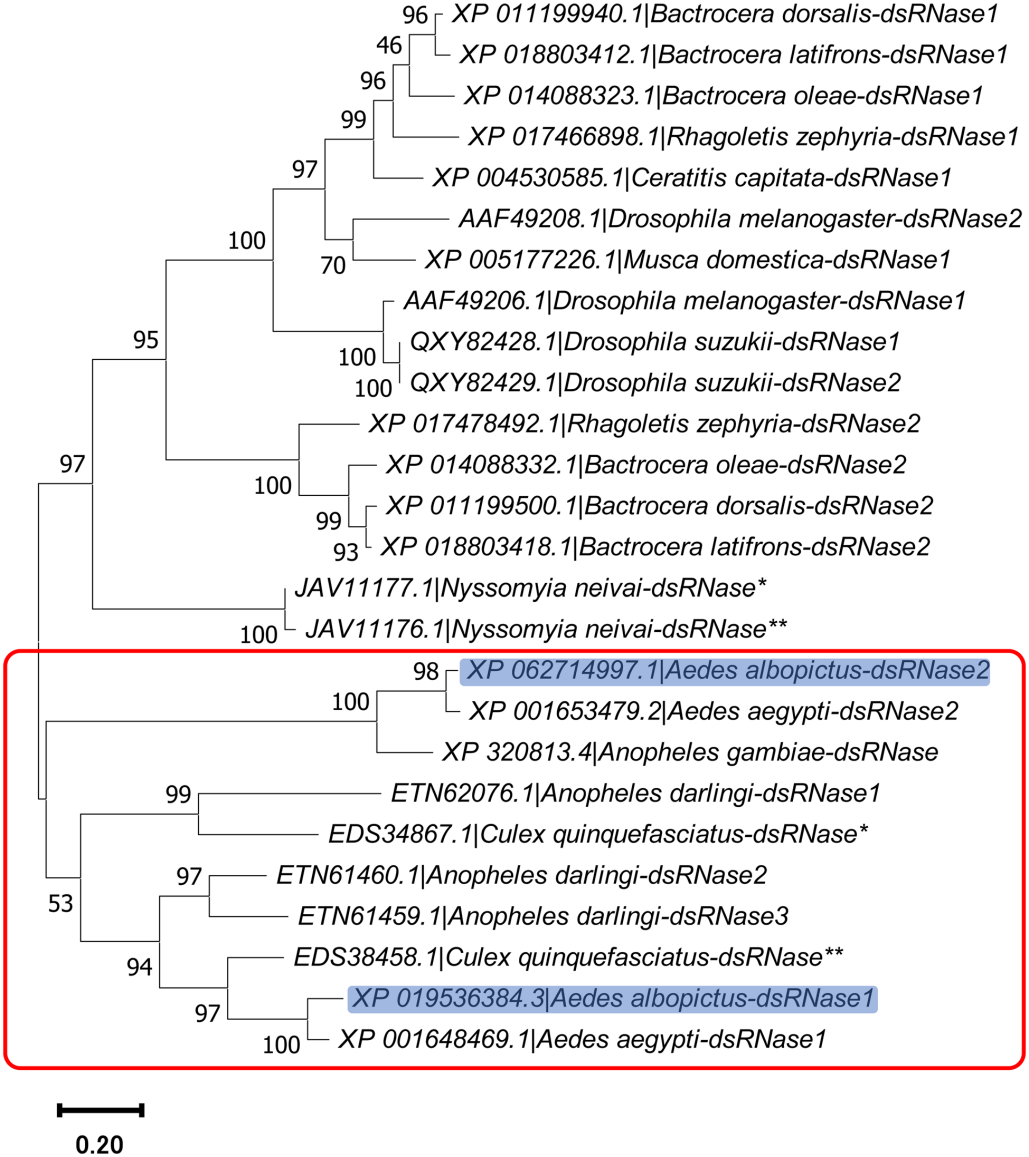
Phylogenetic analysis of dsRNase proteins from dipterans. A phylogenetic tree of 26 dsRNase proteins from 14 species was constructed using the Maximum likelihood method based on the Poisson correction model, with bootstrap replicate (1000 replicates) for the confidence value of each branch (%). The accession numbers are displayed alongside the species names. Asterisks (*, **) distinguish unnumbered proteins within the same species. The complete table for the construction of this tree can be found in Table S2. The red box encompasses the dsRNases of mosquitoes and highlighted in blue are the dsRNases of *Aedes albopictus* reported in this study

The degradation of dsRNA observed in L4 larvae gut extract implies the activity of dsRNases. Therefore, we identified two dsRNases of *Ae. albopictus *via BLASTp search using dsRNase genes from *Ae. aegypti* [28] as queries. Highly related genes were identified, henceforth referred to in this study as Aal-dsRNase1 and Aal-dsRNase2. The protein sequences from both dsRNases cover 100% of the query, with 87.23% and 94.10% identity for Aal-dsRNase1 and Aal-dsRNase2, respectively, compared with *Ae. aegypti* (Table 2). Protein features analysis revealed the DNA/RNA nonspecific endonuclease domains (PF01223) of both dsRNases along with active-, substrate binding-, and Mg^2+^ binding sites (Fig. 7a). Given the ubiquity of dsRNases across the insect taxa [31], understanding their phylogenetic relationships in dipterans is of interest. Therefore, phylogenetic analysis of 26 dsRNase proteins from 14 dipteran species revealed that both dsRNases of *Ae. albopictus* are most closely related to those of *Ae. aegypti* (Fig. 8; Supplementary Information Table S4).

**Table 2:** Identification of *Aedes albopictus* dsRNases

Protein	Protein ID	EPL (aa)	BLASTp			
			Query (*Aedes aegyti*)	Query cover (%)	Identity (%)	E-value
Aal-dsRNase1	XP_019536384.3	415	XP_001648469.1	100	87.23	0.0
Aal-dsRNase2	XP_062714958.1	390	XP_001653479.2	100	94.10	0.0

The dsRNase were retrieved by BLASTp sequence similarity search using *Aedes aegypti* dsRNase 1 and 2 as queries

*EPL* Encoded Protein Length, *aa* amino acid

To better understand the developmental and tissue-specific expression of Aal-dsRNase1 and Aal-dsRNase2, we quantified their expression levels across all four larval stages (L1–L4) using RT-qPCR. The expression of Aal-dsRNase1 was most abundant in L1, followed by L3, L2, and L4 (Fig. 7b). While the expression of Aal-dsRNase2 was highest in L3 followed by L2, L1, and L4. Their expression was also quantified in the dissected gut and rest body of the L4 larvae, where the gut had higher expression of both dsRNases than the rest body (Fig. 7c). To ascertain the accuracy of the amplified targets, we used RT-PCR to amplify Aal-dsRNase1 and Aal-dsRNase2 genes and the products were sequenced and aligned to each respective dsRNase (Supplementary Information, Fig. S5).

### Protection of dsRNA from degradation by *Aedes albopictus* gut extract using commercially available transfection reagents

**Fig. 9:**
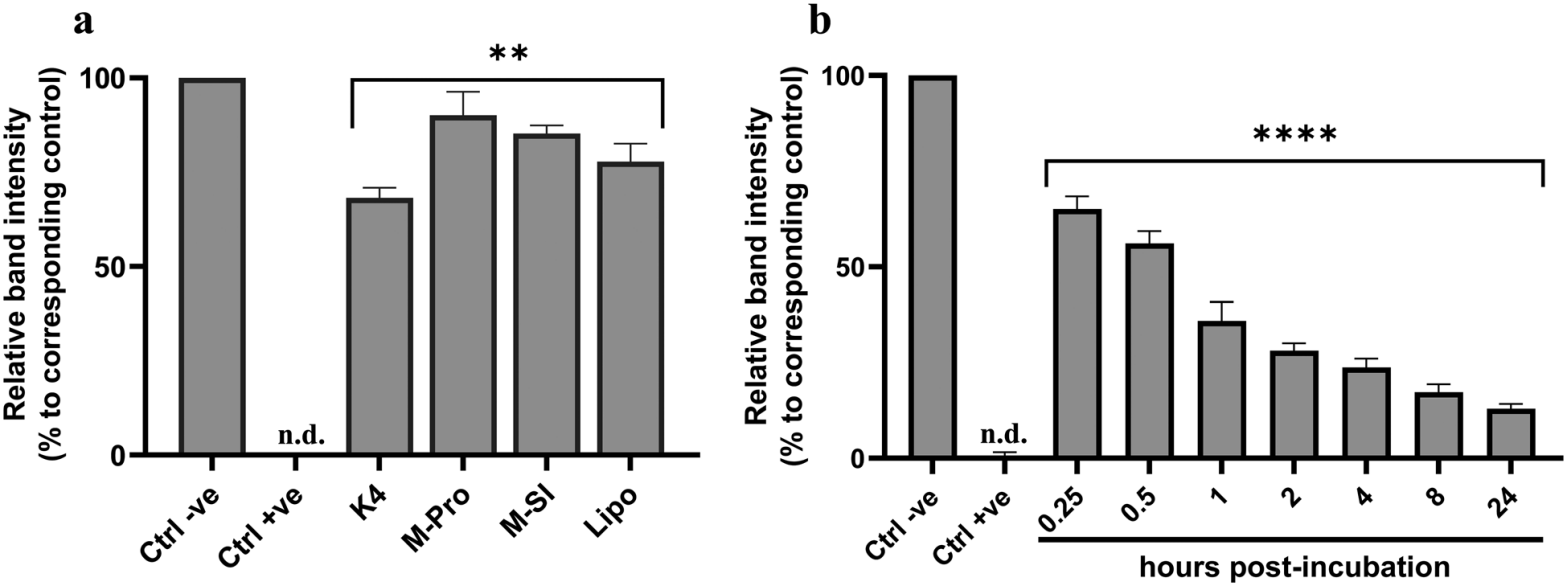
Protection of dsRNA from degradation by *Aedes albopictus* L4 gut extract using transfection reagents. mCherry dsRNA was complexed in **a** with K4 Transfection System (K4), Metafectene Pro (M-Pro), Metafectene SI + (M-SI), or Lipofectamine 2000 (Lipo) and incubated with gut extract for 10 min. In **b**, dsRNA was complexed with K4 and incubated with gut extract for 0.25 to 24 h. EDTA was added post-incubation to stop degradation, and samples were analyzed by agarose gel electrophoresis. Negative control (Ctrl −ve) was incubated in nuclease-free water while positive control (Ctrl + ve) was incubated in gut extract. Gut extract containing EDTA served as independent control for each dsRNA:TR sample and time point. Band intensity was quantified and normalized to controls. Data represent the normalized mean (%) of three independent experiments (*n* = 3), with error bars showing standard deviation. *n.d.* not detectable

To assess whether the TRs could protect dsRNA from degradation, mCherry dsRNA was complexed with four TRs and incubated with L4 larvae gut extract. All TRs provided protection, with Metafectene Pro offering the highest (90%), followed by Metafectene SI + , Lipofectamine 2000, and K4 (Fig. 9a). Since K4 was used in the larval bioassay, we further tested its protective effect over time. K4-complexed dsRNA retained 65% integrity at 0.25 h but decreased to 13% after 24 h (Fig. 9b, the agarose gel images can be found in Supplementary Information Fig. S6).

## Discussion

RNAi is a promising tool for the control of insect vectors and agricultural pests, offering high specificity, minimal off-target effects, and strong environmental safety due to degradation of dsRNA outside the target organism [32, 33]. However, degradation within the larval gut can severely limit efficacy. In this study, we quantify key barriers to oral RNAi in *Ae. albopictus* larvae, showing that gut extracts rapidly degrade long dsRNA, leading to identifying two putative dsRNases potentially responsible for degradation. This study identified the key challenges associated with oral administration and established the design criteria required for more complex formulations to enable the efficient transport of target dsRNA across the epithelial barrier.

Target genes were selected on the basis of previous RNAi studies in other insect pests and vectors, including *Ae. aegypti* [14, 15] and *T. castaneum* [16]. Priority was given to genes associated with high mortality, such as β-tub, Dre4, IAP, ROP, and NCM. Gene-specific dsRNAs were designed using the si-Fi21 tool to ensure high silencing efficiency and minimal off-target effects [24]. Initial screening in *Ae. albopictus* U4.4 cells showed that only IAP 2 dsRNA significantly reduced cell viability, both when delivered alone or complexed with a TR (Fig. 1). Three TRs were tested, including K4, Metafectene Pro, and CellFectin II, showing no substantial difference in their efficacy. To confirm specificity, we used RNAi-deficient C6/36 cells, which lack a functional RNAi pathway due to impaired Dicer-2 function [25, 26]. No cytotoxic effects were observed. RT-qPCR further validated these findings, confirming significant gene knockdown for all tested dsRNAs (Fig. 2). It remains unclear why only one IAP dsRNA variant reduced cell viability. This may be influenced by the biological role of the target gene, compensatory cellular mechanisms, or differences in mRNA structure affecting accessibility [34, 35].

The dsRNAs were further tested in larvae for mortality and gene knockdown. No significant mortality was observed, although gene knockdown occurred in the gut but not in whole larvae. This discrepancy may be due to mRNA dilution from unaffected tissues. Additionally, gut-specific knockdown may trigger compensatory upregulation in other tissues [35]. Meanwhile, K4 alone exhibited significant toxicity at 50 ng/µL, indicating a narrow safety window. Consequently, any potential mortality observed with this TR at that concentration may be difficult to interpret. This lack of mortality was unexpected, particularly for IAP 2 dsRNA, which reduced viability in U4.4 cells and achieved knockdown in both cells and larvae gut. To explore possible limitations related to delivery, we examined dsRNA:TR complex sizes and uptake. At lower concentrations, particle sizes were within the optimal range for endocytic internalization in insect cells, which typically occurs with particle size 100–200 nm [36]. However, a higher concentration resulted in much larger particles (up to 5192.2 nm) with high PIs values, indicating aggregation and broad size distribution [37]. Fluorescence microscopy confirmed dsRNA uptake into the gut, with stronger and more sustained signals for the complexed form (Fig. 5). However, the signal did not spread to the rest of the body. These findings suggest that the large particle size and nonsystemic spread are major contributors to the lack of significant mortality. Although *Ae. albopictus* U4.4 cells can transfer RNAi signal to neighboring cells in vitro [38], systemic RNAi in vivo appears limited. Following oral delivery, knockdown was restricted to the midgut, indicating the need for delivery approaches that enhance dsRNA stability in the gut and enable transcytosis across the midgut epithelium.

To determine whether dsRNA stability limits RNAi efficacy in *Ae. albopictus* larvae, we investigated its degradation in the gut environment. Complete degradation was observed within 4 min (Fig. 3a), much faster than in other insects. For example, only 77% of GFP dsRNA was degraded after 2 h in gut of the workers of tawny crazy ant, *Nylanderia fulva* [21]. The inhibition of degradation by EDTA (Fig. 6b) indicates that the responsible nucleases are metal ion-dependent. This led to the identification of two dsRNases (Aal-dsRNase1 and Aal-dsRNase-2), containing a signal peptide and a nonspecific endonuclease domain suggesting a role in extracellular degradation [21, 28, 39]. Their predicted Mg^2+^ binding sites (Fig. 7a) also supports their observed metal ion dependency. Phylogenetic analysis revealed close clustering with other mosquito dsRNases (Fig. 8). Developmental expression of both dsRNases declined in L4, likely owing to reduced feeding and metabolic shifts before pupation [40], but remained significantly higher in the gut than in the rest of the body (Fig. 7c). This expression pattern is consistent with observations from other insects [12, 21, 28, 41]. These findings suggest that rapid dsRNA degradation and high gut expression of dsRNases are major barriers to RNAi in larvae. Nevertheless, IAP 2 dsRNA achieved significant gut-specific knockdown, indicating that nuclease activity alone does not explain the limited efficacy. Additional factors such as endosomal degradation, suboptimal target selection, insufficient dose or exposure, and microbiota interactions are likely involved [42]. Therefore, achieving lethal effects in larvae requires addressing multiple biological hurdles beyond nuclease protection.

We assessed whether complexation could protect dsRNA from gut degradation. While it offered temporary protection, its effectiveness declined over time (Fig. 9), suggesting that dsRNA degradation in the gut may contribute to the lack of RNAi-induced mortality. Although, silencing dsRNase genes has improved RNAi efficacy in *Ae. aegypti* and other insects [40, 43, 44], this strategy may be ineffective here due to rapid degradation of the dsRNA itself. For instance, previous attempts to co-deliver short hairpin RNA (shRNA) against gut nucleases in *Ae. aegypti* and *Ae. albopictus* also did not enhance RNAi [19].

RNAi effects observed in U4.4 cells may not accurately reflect in vivo conditions, as the cell line originates from whole neonate *Ae. albopictus* larvae rather than specifically from gut tissue [45]. Consequently, dsRNA is not subjected to digestive or nuclease activity, microbiota interactions, and immune factors. While U4.4 cell model remains valuable for mechanistic screening, primary midgut cultures or ex vivo gut sacs would provide more physiologically relevant epithelial models, making them more suitable for target genes with pronounced tissue-dependent expression [46]. To the best of our knowledge, no study has reported significant mortality in *Ae. albopictus* larvae using uncomplexed dsRNA alone. However, in *Ae. aegypti*, uncomplexed dsRNA has successfully induced gene knockdown and mortality when targeting genes such as HSP83, β-tubulin, voltage-gated sodium channel, or chitin synthases A/B [15, 47, 48], highlighting a clear interspecies disparity in RNAi sensitivity. The enhanced RNAi efficacy observed in *Ae. aegypti* following dsRNase gene silencing may result from reduced dsRNase expression and activity, differences in the functionality of the core RNAi machinery, or variations in immune gene complexity, as *Ae. albopictus* possesses a larger genome with extensive gene duplications [43, 49–51]. While these factors could lead to interspecies difference in RNAi responsiveness, the molecular basis and direct evidence linking them are still lacking. Nevertheless, significant RNAi-induce mortality has been achieved in *Ae. albopictus* when using engineered *Saccharomyces cerevisiae* expressing shRNAs targeting synaptotagmin, ataxin-2-binding protein, or semaphoring-1a [52–54]. Therefore, their findings indicate that the gut barrier can be overcome, but effective RNAi will require an efficient delivery system.

Meanwhile, classical RNAi approaches without genetic modification are more compatible with current biosafety regulations [7, 55]. Nonetheless, the inconsistent RNAi response in *Ae. albopictus* remains a major challenge that must be overcome [19]. Given that the gut is the primary site of oral dsRNA uptake and IAP gene knockdown was achieved in the larval gut, future studies should focus on identifying gut-specific essential genes in *Ae. albopictus*. Our data identify key quantitative barriers to oral RNAi in *Ae. albopictus* larvae, driven by rapid degradation of dsRNA by gut nucleases, suboptimal complex size, and limited systemic spread. Consequently, delivery is the key determinant of efficacy, exceeding the influence of dsRNA design or dose. Promising delivery platforms include yeast-based delivery platforms, lipid vesicles, polymeric nanoparticles, biodegradable polyplexes, virus-like particles specific to mosquitoes, and inorganic hybrids [14, 54, 56–59]. Such formulations must protect dsRNA from early degradation in the gut by nucleases, maintain optimal particle sizes for efficient oral uptake, promote effective epithelial translocation, and provide controlled intracellular release. Additional strategies for improving stability may include chemical modification like a phosphorothioate backbone or co-delivery of species-specific nuclease inhibitors to reduce gut nuclease activity [60].

For field-ready larvicides, the product should pair effective formulations with deployment practicality. Target selection should prioritize gut-specific essential genes, ideally in multi-gene combinations to mitigate potential resistances, and efficacy should be validated under realistic environmental conditions. Development pipelines should incorporate laboratory, mesocosm, and pilot-scale trials to confirm persistence, safety, and scalability. Ultimately, a functional RNAi-based larvicide will rely on optimized formulations that ensure dsRNA stability, uptake, and reproducible efficacy, together with full regulatory compliance [7, 61].

## Conclusions

This study highlights the potential and limitations of RNAi for controlling *Ae. albopictus*. Only one dsRNA construct induced cytotoxic effects in cell-based assays, despite all selected dsRNAs achieving significant gene knockdown. In larval assays, none of the dsRNAs induced mortality, despite a significant gene knockdown in the gut. Limited RNAi efficacy in the larvae may be as a result of insufficient systemic spread of dsRNA, poor cellular internalization, and its rapid degradation by gut nucleases. These findings emphasize the complexity of translating gene silencing into functional lethality in whole organisms, by specifically providing a quantitative degradation of the dsRNA, as well as molecular identification and characterization of *Ae. albopictus* dsRNases. Further research is needed to clarify the molecular basis of reduced RNAi sensitivity in *Ae. albopictus* compared with *Ae. aegypti*. Most importantly, identifying gut-specific essential genes, exploring suitable formulations, and understanding species-specific barriers, is crucial for realizing RNAi’s full potential as a control method against *Ae. albopictus*.

## Supplementary Information

Additional file 1.

## Data Availability

All data generated or analyzed during this study are included in this published article.
