# Supplementary material for: RNA interference mediated mortality in Aedes albopictus: a challenging journey toward species-specific vector control
Source: Parasit Vectors. 2025 Nov 14;18:463. doi: 10.1186/s13071-025-07113-2 (PMC12619383; doi:10.1186/s13071-025-07113-2)
Supplement: Supplementary file 1 — Additional file 1. [file 13071_2025_7113_MOESM1_ESM.docx]

**Supplementary Information**

**RNA interference mediated mortality in *Aedes albopictus*: a challenging journey towards species-specific vector control**

**Bodunrin Omokungbe^1,2,3^, Alejandra Centurión^1,3^, Sabrina Stiehler^2^,** **Magnus Wolf^4,5^, Pascal Geisler^3^, Andreas Vilcinskas^1,2,3^, Antje Steinbrink^1,2^, Kornelia Hardes^1,3,4,*^**

^1^ LOEWE Centre for Translational Biodiversity Genomics (LOEWE TBG), Senckenberganlage 25, 60325 Frankfurt am Main, Germany

^2^ Institute for Insect Biotechnology, Justus-Liebig University, Heinrich-Buff-Ring 26–32, 35392 Giessen, Germany

^3^ Fraunhofer Institute for Molecular Biology and Applied Ecology IME, Branch of Bioresources, Ohlebergsweg 12, 35392 Giessen, Germany

^4^ Institute for Evolution and Biodiversity (IEB), University of Muenster, Huefferstrasse 1, 48149, Muenster, Germany

^5^ Senckenberg Biodiversity and Climate Research Centre (BiK-F), Georg-Voigt-Strasse 14-16, 60325, Frankfurt am Main, Germany

^6^BMBF Junior Research Group in Infection Research “ASCRIBE” Ohlebergsweg 12, 35392 Giessen, Germany

* Correspondence: [Kornelia.Hardes@ime.fraunhofer.de](mailto:kornelia.Hardes@ime.fraunhofer.de)

## **1 Candidate gene target selection, double-stranded RNA design and synthesis**

To identify suitable RNAi target genes in *Aedes albopictus*, we conducted a literature search to compile genes previously associated with high mortality in other insect species. For each selected gene, two dsRNA constructs were designed and synthesized. The sequences of the primers used for dsRNA synthesis, gene knockdown validation, and analysis of dsRNase expression profiles are listed in Table S1.

**Table S1**: Primer sequences used for dsRNA synthesis, validation of gene knockdown, and expression profiles of dsRNases by RT-qPCR. The T7 promoter sequence (in lower case) was added to the 5′ end of each primer used for dsRNA synthesis. RT-qPCR primers for confirming gene knockdown were designed to bind near the 5′ region of the target gene, outside the dsRNA target sequence.

| Gene | purpose | Name of fragment | Primer sequence 5’ 🡪 3’ | size (bp) |
| --- | --- | --- | --- | --- |
| Green fluorescent protein (GFP) | RNAi | gfp484_T7_fw | ccctttaatacgactcactatagggagaACCACATGAAGCAGCACGACTT | 484 |
|  |  | gfp484_T7_rv | ccctttaatacgactcactatagggagaGTCCATGCCGAGAGTGATCCCG |  |
| mCherry | RNAi | mCh_T7-fw | taatacgactcactatagggGCGTGATGAACTTCGAGGAC | 409 |
|  |  | mCh_T7-rv | taatacgactcactatagggCTTGTACAGCTCGTCCATGC |  |
| β-Tubulin (*β-tub*) | RNAi | β-tub-1_T7-fw | taatacgactcactatagggGTCGACGAACAGATGCTGAA | 494 |
|  |  | β-tub-1_T7-rv | taatacgactcactatagggAAGTGGCGCAGATAGAGGAA |  |
|  |  | β-tub-2_T7-fw | taatacgactcactatagggCACGACATGGACGTTACCTG | 496 |
|  |  | β-tub-2_T7-rv | taatacgactcactatagggGGGGGAGAAGGGTACAGAAG |  |
|  | RT-qPCR | β-tub-1_fw | GGTTTTCTCGTGTTCCGTCG | 221 |
|  |  | β-tub-1_rv | GCGTCGATTCCATGCTCATC |  |
| Dre4 (D*re4*) | RNAi | Dre4_T7-1_fw | taatacgactcactatagggTTTCGGTGAAGTTTTCGGGC | 417 |
|  |  | Dre4_T7-1_rv | taatacgactcactatagggCCTCCTCGGCATCCTTCTCTA |  |
|  |  | Dre4_T7-2_fw | taatacgactcactatagggATCCGGCCATTATCCAGTCG | 446 |
|  |  | Dre4_T7-2_rv | taatacgactcactatagggTGCCTCCTTGTTCTCCAAGC |  |
|  | RT-qPCR | Dre4_PCR_fw | CGGTCCCATCATAGTTCGTCC |  |
|  |  | Dre4_PCR_rev | ATGCGTCACTTCCTCCAGTT |  |
| Ras Opposite (*ROP*) | RNAi | ROP-1_T7-fw | taatacgactcactatagggTGAGGTGTGCCCTGAAGAAC | 495 |
|  |  | ROP-1_T7-rv | taatacgactcactatagggGCGTTCGGAGATGGGATGAA |  |
|  |  | ROP-2_T7-fw | taatacgactcactatagggGATTGCGTTTCTCCGTTGCT | 447 |
|  |  | ROP-2_T7-rv | taatacgactcactatagggGCGCATGTGGTCCTTGATTT |  |
|  | RT-qPCR | ROP _fw | CTGGGTATCAACGTCATCGCT | 243 |
|  |  | ROP_rv | TGACTTGTCCTTGTGCCAGT |  |
| Inhibitor of apoptosis (*IAP*) | RNAi | IAP-1_T7-fw | taatacgactcactatagggCGTTGTGTGGTCGGTCTAGT | 403 |
|  |  | IAP-1_T7-rv | taatacgactcactatagggATCTTGAGTTCGCGGCTGTT |  |
|  |  | IAP-2_T7-fw | taatacgactcactatagggACACCGGCAAAAGTGATCGT | 425 |
|  |  | IAP-2_T7-rv | taatacgactcactatagggACAGGGCGAAAATGCCGTAT |  |
|  | RT-qPCR | IAP_ fw | GGCTCAAACGATGATGGCAC | 173 |
|  |  | IAP_rv | TCTTGCTGATGAAGGGCACC |  |
| Nucampholin (*NCM*) | RNAi | NCM-1_T7-fw | taatacgactcactatagggGATTCCCGTTTCGACTCCGA | 449 |
|  |  | NCM-1_T7-rv | taatacgactcactatagggTTGTCCGTAATCTCCGCCTG |  |
|  |  | NCM-2_T7-fw | taatacgactcactatagggTCGTCGGAAAAAGAAAGGCA | 408 |
|  |  | NCM-2_T7-rv | taatacgactcactatagggTCTACGGTCACAATACAGGGAA |  |
|  | RT-qPCR | NCM_ fw | GCGGGGCTATGAAAGAGAGG | 222 |
|  |  | NCM_rv | CTCCGGCGACTAGAGGAAGA |  |
| Actin | RT-qPCR | Actin_fw | AGATCCTGACTGAACGTGGC | 162 |
|  |  | Actin_rv | CGTCGGGAAGTTCGTAGGAC |  |
| Aal-dsRNase1 | RT-qPCR | Aal-dsRNase1-fw | ATTCGATTTGGGAACCCGCT | 237 |
|  |  | Aal-dsRNase1-rv | GCGAGTTCTTGCGATTGGAG |  |
| Aal-dsRNase2 | RT-qPCR | Aal-dsRNase2-fw | ACCCGAAAAATTGCGCTACG | 204 |
|  |  | Aal-dsRNase2-rv | AGATGGTGGTAGTCTCGCCA |  |

## **2 Cytotoxicity effects of RNAi in aedine cell lines**


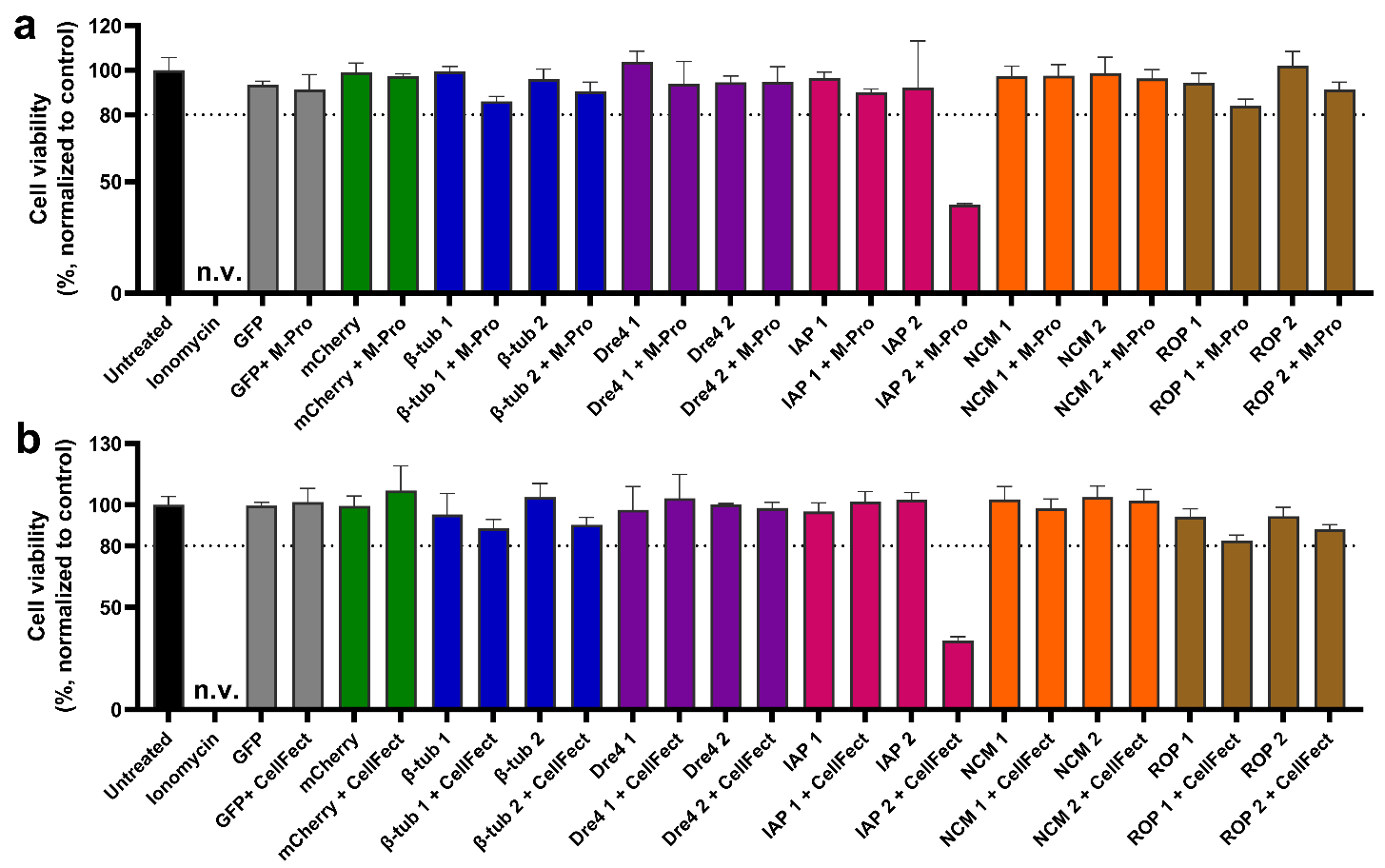


**Fig. S1.** Cytotoxic effects of complexed dsRNAs in *Aedes albopictus* U4.4 cells. Cells were treated at ~50% confluency with dsRNA at a concentration of 2 ng/µL per well (n = 4). The dsRNAs were complexed in (**a**) with Metafectene Pro (M-Pro) and in (**b**) CellFectin II (CellFect) before treatment. At 48 h post-treatment cell viability was assessed using the CellTiter-Glo assay. Data were normalized to the untreated control (treatment/control × 100), and mean cell viability is shown. Error bars represent the coefficient of variation (%). The dotted line indicates the toxicity threshold set at 80%. n.v. = near-zero viability.

**3 Gene silencing analysis in U4.4 cells and larvae of *Aedes albopictus***

Prior to gene silencing analysis, a standard curve analysis (0.1–1000 ng) was performed for each primer pair to determine efficiency, using total RNA extracted from L4 larvae with the Monarch Total RNA Miniprep Kit (New England Biolabs, Frankfurt, Germany). RNA concentration and purity were assessed using a NanoDrop 2000 spectrophotometer (Thermo Fisher Scientific, Frankfurt, Germany). RT-qPCR was performed with the Luna Universal One-Step RT-qPCR Kit (New England Biolabs) on a QuantStudio 3 Real-Time PCR System (Applied Biosystems, Thermo Fisher Scientific). Detailed reaction components and thermal cycling conditions are provided in Table S2. Primer’s efficiencies are shown in Fig. S2, and the gene knockdown results in whole larvae are presented in Fig. S3.

**Table S2**: Reaction mix and thermal program for RT-qPCR for gene knockdown and expression profile analysis.

| **Reaction mix** | | | |
| --- | --- | --- | --- |
| **Reaction component** | | | **Volume (µL)** |
| Luna Universal One-Step Reaction Mix (2x) | | | 10.0 |
| Luna WarmStart RT Enzyme Mix (20x) | | | 1.0 |
| Gene-specific Forward Primer (10 µM) | | | 0.8 |
| Gene-specific Reverse Primer (10 µM) | | | 0.8 |
| Nuclease-free Water | | | 2.4 |
| Template RNA (20ng/µL) | | | 5.0 |
| **Total Volume** | | | **20.0** |
| **Thermal parameter** | | | |
| **Cycle step** | **Temperature** | **Time** | **Cycles** |
| Reverse Transcription | 55°C | 10 min | 1 |
| Initial Denaturation | 95°C | 1 min | 1 |
| Denaturation | 95°C | 10 sec | 45 |
| Extension | 60°C | 60 sec |  |
| Melt Curve | 60-95°C | various | 1 |


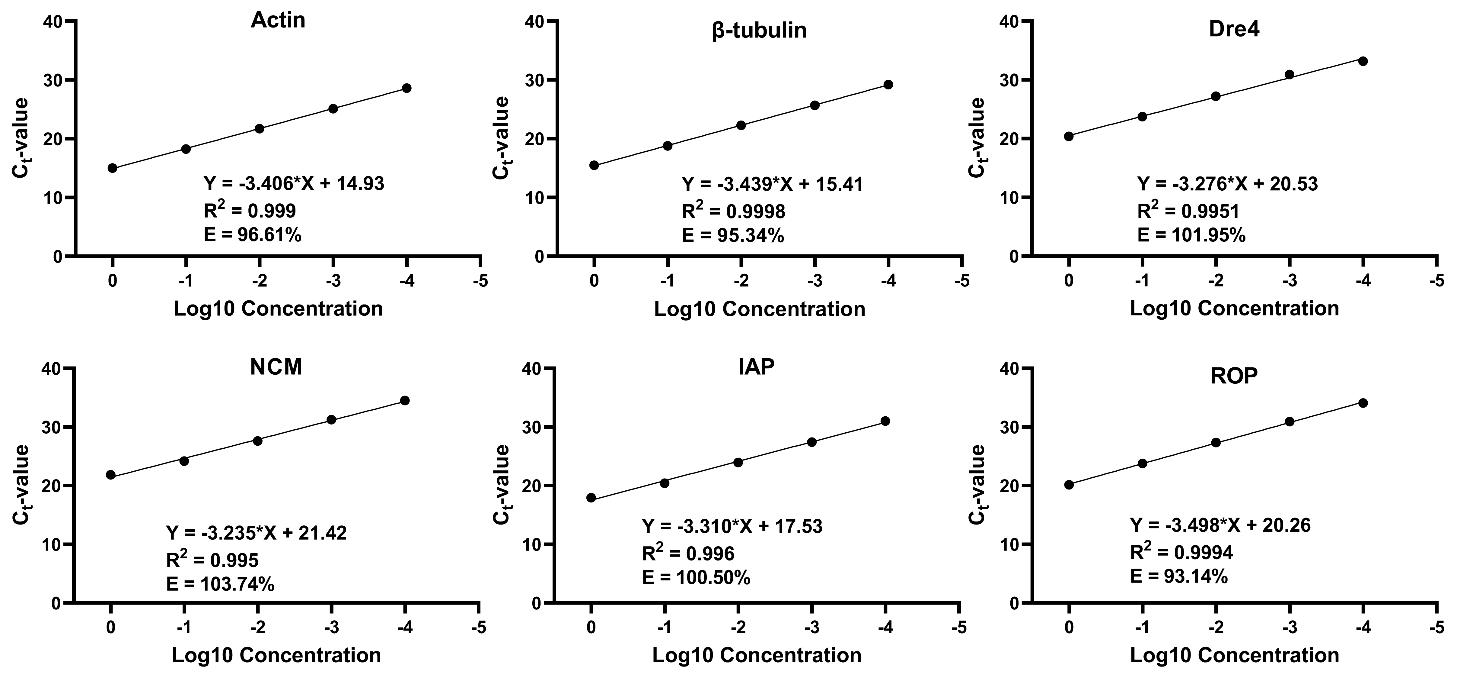


**Fig. S2**. Primer efficiency of RT-qPCR primers. The efficiency was assessed *via* the standard curve analysis using total RNA extracted from L4 larvae (0.1–1000 ng). C_t_ values were plotted against the logarithm of RNA concentrations to generate standard curves. The slope, Y-intercept, coefficient of determination (R²), and primer efficiency (E) are indicated for each primer set.


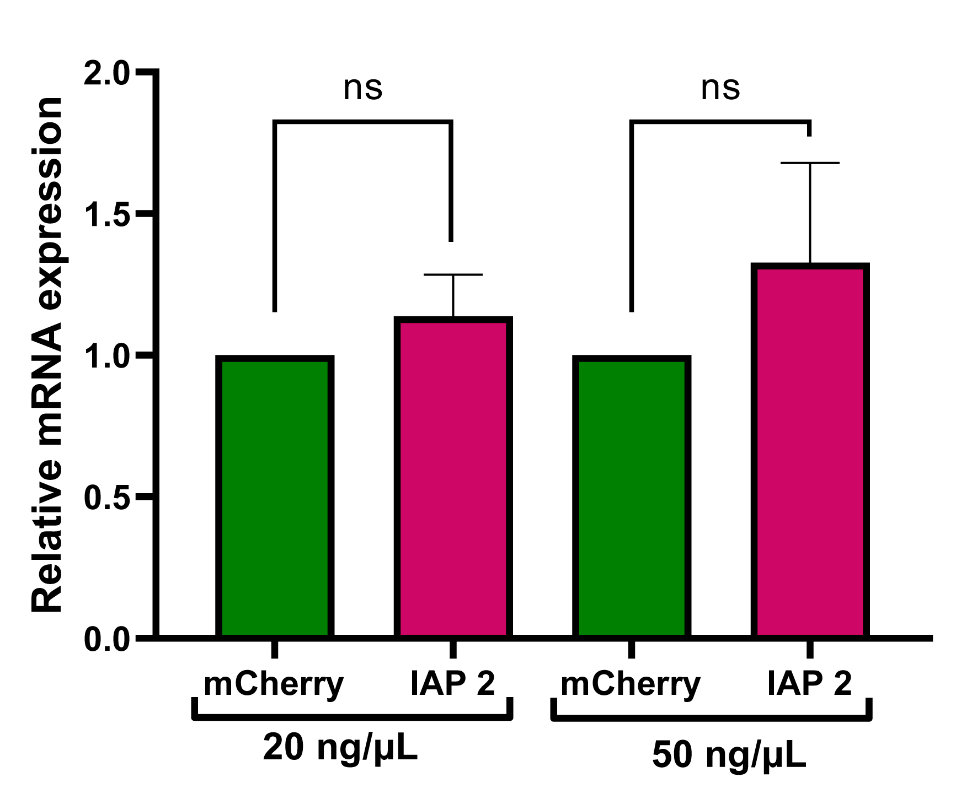


**Fig. S3.** Gene knockdown in whole larvae following treatment with complexed IAP 2 dsRNA using the K4 Transfection System (1:1). Larvae were treated using the second bioassay approach described in the main manuscript, and at 3 days post-treatment RNA were extracted for RT-qPCR analysis. Actin was used as the reference gene, and relative expression was calculated using the 2^−ΔΔCt^ method, normalized to the non-specific mCherry dsRNA control (n = 3). Error bars represent standard deviation. ns = no significant difference (p > 0.05), determined by one-way ANOVA and Šidák’s multiple comparison test.

**4 Dynamic light scattering analysis of formulated dsRNA**

Since the dsRNAs did not caused mortality in larvae, we tested whether the size of the complexes was a limiting factor. Therefore, we performed dynamic light scattering (DLS) to determine the particle size of dsRNA:TR complexes. First, the mCherry dsRNA was complexed with either K4 (1:1), Metafectene Pro (1:0.7), or CellFectin II (1:5) to a final concentration of 10, 20, or 50 ng/µL. The complexes were incubated at room temperature for 20 min. Nuclease-free water was used as the base medium for all dilutions, instead of the recommended Grace’s insect medium, to match the conditions used in the larval experiments. For particle size measurements, 5 µL of each complex was transferred into the capillary tube of a Zetasizer Low Volume Disposable Sizing Cell Kit (ZSU1002, Malvern Panalytical, Malvern, United Kingdom) and analyzed using a Malvern Ultra Red Zetasizer (Malvern Panalytical). Particle size was analyzed using the side scattering method. The results are shown in Table S3.

**Table S3.** Particle size distribution of dsRNA:TR complexes. mCherry dsRNA was complexed with K4 Transfection System, Metafectene Pro, or CellFectin II, and particle size was analyzed using dynamic light scattering with side scattering method. The sample size was n=3 and each sample replicate were measured three times.

| **Sample** | **Concentrations (ng/µL)** | **Particle size (Z-Average nm)** | **Polydispersity Index (PI)** |
| --- | --- | --- | --- |
| K4 Transfection System | 10 | 155.3 | 0.1018 |
|  | 20 | 2461.7 | 0.5107 |
|  | 50 | 5192.2 | 0.5426 |
| Metafectene Pro | 10 | 150.6 | 0.1348 |
| CellFectin II | 10 | 165.8 | 0.1776 |

**5 Ex vivo degradation of dsRNA using *Aedes albopictus* gut extract**


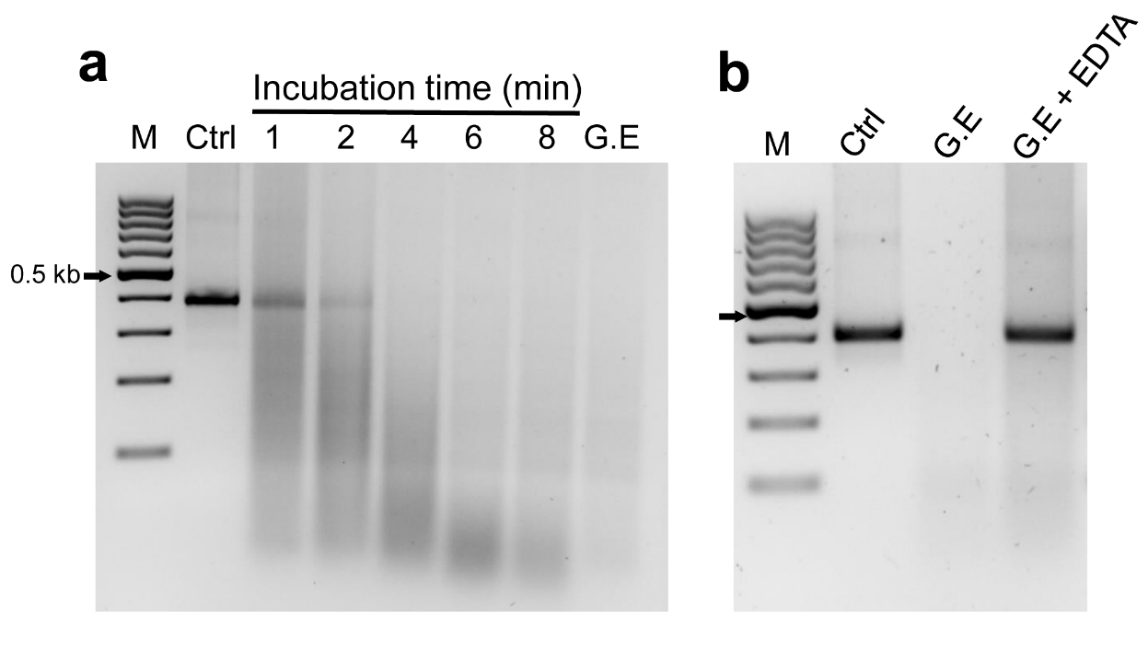


**Fig. S4**. Ex vivo degradation of dsRNA with gut extract of *Aedes albopictus* larvae. The gut extract from L4 larvae was used for the incubation of mCherry dsRNA. In (**a**), the dsRNA was incubated with gut extract for 1 – 8 min. In (**b**), the dsRNA was incubated for 10 min with either gut extract or gut extract containing EDTA. All samples were resolved on agarose gel electrophoresis immediately after each incubation and a GeneRuler 100 bp DNA ladder (M, Thermo Fisher scientific) was used as marker. The gel images displayed a representative of three independent experiments (n=3). Ctrl= mCherry dsRNA incubated in nuclease free water, G.E = gut extract alone, while G.E + EDTA = mixture of gut extract and ethylenediaminetetraacetic acid.

**6 Identification, characterization, and expression profile of *Aedes albopictus* dsRNases**

**Table S4**: List of dsRNase proteins used for the construction of the phylogenetic tree. The accession number for the nucleotide sequence were recovered from literature, quality-checked and the translated protein sequence were retrieved from NCBI (see Table S5 below). The asterisk (*,**) denotes multiple dsRNases within the same species

| **Species** | **dsRNase** | **Accession number** | **References** |
| --- | --- | --- | --- |
| *Aedes aegypti* | Aae-dsRNase1 | XP_001648469.1 | Yoon et al 2021 |
|  | Aae-dsRNase2 | XP_001653479.2 | Yoon et al 2021 |
| *Aedes albopictus* | Aal-dsRNase1 | XP_019536384.3 | This study |
|  | Aal-dsRNase2 | XP_062714997.1 | This study |
| *Anopheles darlingi* | Ad-dsRNase1 | ETN62076.1 | Cooper et al 2020 |
|  | Ad-dsRNase2 | ETN61460.1 | Cooper et al 2020 |
|  | Ad-dsRNase3 | ETN61459.1 | Cooper et al 2020 |
| *Anopheles gambiae* | Ag-dsRNase | XP_320813.4 | Cooper et al 2020 |
| *Bactrocera dorsalis* | Bd-dsRNase1 | XP_011199940.1 | Sharma et al 2021 |
|  | Bd-dsRNase2 | XP_011199500.1 | Sharma et al 2021 |
| *Bactrocera latifrons* | Bl-dsRNase1 | XP_018803412.1 | Tayler et al 2019 |
|  | Bl-dsRNase2 | XP_018803418.1 | Tayler et al 2019 |
| *Bactrocera oleae* | Bo-dsRNase1 | XP_014088323.1 | Tayler et al 2019 |
| *Ceratitis capitata* | Cc-dsRNase1 | XP_004530585.1 | Tayler et al 2019 |
|  | Bo-dsRNase2 | XP_014088332.1 | Tayler et al 2019 |
| *Culex quinquefasciatus* | Cq-dsRNase* | EDS34867.1 | Cooper et al 2020 |
|  | Cq-dsRNase** | EDS38458.1 | Cooper et al 2020 |
| *Drosophila melanogaster* | Dm-dsRNase1 | AAF49206.1 | Cooper et al 2020 |
|  | Dm-dsRNase2 | AAF49208.1 | Cooper et al 2020 |
| *Drosophila suzukii* | Ds-dsRNase1 | QXY82428.1 | Yoon et al 2021 |
|  | Ds-dsRNase2 | QXY82429.1 | Yoon et al 2021 |
| *Musca domestica* | Mm-dsRNase1 | XP_005177226.1 | Tayler et al 2019 |
| *Nyssomyia neivai* | Nn-dsRNase* | JAV11177.1 | Cooper et al 2020 |
|  | Nn-dsRNase** | JAV11176.1 | Cooper et al 2020 |
| *Rhagoletis zephyria* | Rz-dsRNase1 | XP_017466898.1 | Tayler et al 2019 |
|  | Rz-dsRNase2 | XP_017478492.1 | Tayler et al 2019 |

**Table S5**: List of protein sequence used for the construction of the phylogenetic tree.

| 1 | >XP_019536384.3\|*Aedes albopictus*-dsRNase1  MEKFTCLMTILVLLGAGSDSKEIPSITSDNQRFAPSCSMNINNQLPRPQPLLLIPGTEEFRYPATSNRQL  RLNPGETIELVCSSGFDLAPNKNSIIVSCVIDTIFNYDSTMHQFTDFSCRQIWYSNARRTYEPCERNASI  IQIGFDLGTRFPKIMDICHNEETFENHWIKHEMFPFNAGYQSSNPRPNWYQGNFYPGIDTNYLYTNNKQR  QTVAQILQSQELADDIIRDVGSGVYMARGHIAARVDFLYGTQQNATFWFLVVAPQWQNFNDGNWLRIEEQ  VREFVAARNINVTVYGGTYGAHTQTDVNGDQQPIFLDYDPNGVQRLPAPKIYYKILHDERHNSGIALIGV  NDIHITSMEQINGQYLLCDDIGDKVSWINWDRRNFKKGFAYACEVNPFLKRIGHLAHLDIPNLLI |
| --- | --- |
| 2 | >XP_062714997.1\|*Aedes albopictus*-dsRNase2  MNRVASLLVLTVALELVWGQCTVNIRTNLVSPEPVFFRTATQLWSPDGPSLFWNSGETTTISCQSGTLTG  FGVSTASLTCQSGTSFTIGGTAVSSSALTCTQRITGEIQTTTTSCGGGAGQLRNIGFLNPSGQMVTYIQS  CYNVNTASVIYTRHIIPGRAINHAIQESYRPSFKVAGTASHVSPATSYTTASQATRLAVLLGSQAQADRF  ITTSSYMSRGHLSPDADGIFRSWQWATYFYVNVAPQWQVVNAGNWLTVEGAARNIAGRLQEDVLIFNGCH  DVMTLPHVNGQQIPITLEAGGIQAPKWYWKIIKSPSTNSGIALITNNDPFRTSMPAAEMLCSDVCSTYGW  ANANYGNFGRGFTYCCTVAALMSAIPAIPAEAAASNVLRY |
| 3 | >QXY82428.1\|*Drosophila suzukii*-dsRNase1  MKGLVLIALSGLFLASGQARILPKEDLPWELPAIPEVVNEIEPREAGCSIKIRSSELKDPQPLLIKSGTS  EIVGFSDSGNVDVDKDKTIEFHCTSSLASPLSGKSVTAKCVGGTTFKIDDKEHDLSAIKCTSWPAFVGKK  SGSSCNGGTTLIKVGFELSGSRFATQYEVCFNEDEEVTRYVYHRLEPGNNYYATGVDRITFGAGGYFAGK  NVDKLYTQAVQKETIDKELDMDSAHYFNSAKNIFLARGHMGAKADFVFAPEQRATFLFINAAPQWQTFNA  GNWARVEDGVRAWVAKEKKHVECWTGVWGVTTLANKNGEQRQLYLSHDKNGNGLIPVPKLYFRVVIEPSS  KKGIVLIGVNNPHLSLEEIKRDYILCTDVSDKIDWISWKKTDLTAGYSYACEVAEFRKKVDHLPEFSVL |
| 4 | >QXY82429.1\|*Drosophila suzukii*-dsRNase2  MKGLVLIALSGLFLASGQARILPKEDLPWELPAIPEVVNEIEPREAGCSIKIRSSELKDPQPLLIKSGTS  EIVGFSDSGNVDVDKDKTIEFHCTSSLASPLSGKSVTAKCVGGTTFKIDDKEHDLSAIKCTSWPAFVGKK  SGSSCNGGTTLIKVGFELSGSRFATQYEVCFNEDEEVTRYVYHRLEPGNNYYATGVDRITFGAGGYFAGK  NVDKLYTQAVQKETIDKELDMDSAHYFNSAKNIFLARGHMGAKADFVFAPEQRATFLFINAAPQWQTFNA  GNWARVEDGVRAWVAKEKKHVECWTGVWGVTTLANKNGEQRQLYLSHDKNGNGLIPVPKLYFRVVIEPSS  KKGIVLIGVNNPHLSLEEIKRDYILCTDVSDKIDWISWKKTDLTAGYSYACEVAEFRKKVDHLPEFSVL |
| 5 | >XP_001648469.1\|*Aedes aegypti*-dsRNase1  MAQFTILVTFLILLGARSESKELPSKVSDNQRFAPSCSININTQLPRPQPLLLIPGTEEFRYPATSNRLL  RLNPGETVELVCSNGFNLAPSKNSIIVSCVIDTVFNYDSTMRQFSDFSCRQIWYSSARRTYEPCENSSSI  IQIGFDLGARFPKIMDICHNEETFENHWIKHEMYAANAGYQSSNPRPNWYQGDFYPGIDTNYLYTVNKQR  QTIAQILQSQDLADDIVRDVNSGIYMARGHIAARVDFIYGTQQNATFWFLVVAPQWQNFNDGNWLRVEEQ  VRVFIAARNLNVTVYGGTYGAHTQTDANGDQQPIFLDYDPNGVQRLPAPKIYYKILHDERNQAGIALVGV  NDIHITSMEQIEEQYMFCEDIGDKVSWINWERRNFKKGFAYACEVNPFLKRIGHLAHLDVPNLLI |
| 6 | >XP_001653479.2\|*Aedes aegypti*-dsRNase2  MIRIGSLFVLAVALELVWGQCTVNLRTNLVSPEPVFFRTATQLWSPDGPSLFWNSGETTTISCQSGTLTG  FGVSTASLTCQSGTSFTIGGIPVSSAALTCSQRITGEIQITSTSCGGGAGQLRNIGFLNPSSQLVTYIQS  CYNVNTASVIYTRHIIPGRAINHAIQESYRPSFKVAGTASHVSPASSYTTASQATRLAVLLGSQAQADRF  ITTSSYMSRGHLSPDADGIFRSWQWATYFYVNVAPQWQVVNAGNWLTVEGAARNIAGRLQEDVLIFNGCH  DVMTLPHVNGQQIPITLEAGGIQAPKWYWKIIKSPNTNSGIALITNNDPFRTSMPAGEMLCQDVCSTYGW  GNANYGNFARGFTYCCTVAALMSAIPAIPSEAAVANVLRY |
| 7 | >XP_011199940.1\|*Bactrocera dorsalis*-dsRNase1  MKLTSTLLLLVAGSFCLFHGCTAGVVAVPEDVVDDWKADDHMSEQINILFERNLYVEPIKEDASQAIVPA  EVEEDYVEPEPISEVQDELVQPDVDVNGKIEGRASECKVTIRGGLPTPQPLYLKSGSEEIYPYDTKGVMV  VDAGSTLEMWCPGKFTTLDTTLVTATCVSGTNFRVDGTTYAFKELTCKAWPTFVAEKTGASCNGGIMVRV  GFKISSTRFAKQYEVCFNEGDEVTRYVHHDLNPGANYYQTGVDRITFQTGGFFDGKNVDKLYTQATQLET  INAHLGGDASKYFDSAKNVYLARGHMAAKADFDYGLEQRATFLFINAAPQWQVFNAGNWARIEDGVRAKV  SSAGWYVDCYTGVYGVTTLANSDGVQTPLYLAYDSNNNGLIPVPKLYFRVVIEKTSKKGIVFIGVNNPHL  TLDEIKKDYILCTDIADQVDYISWKRTDLTAGYSYACEVSDFRSKVTNLPSLSAPGGLLL |
| 8 | >XP_011199500.1\|*Bactrocera dorsalis*-dsRNase2  MYSGGKAVKLALVLACLLAVVEAGILHSKETAVKSLLPNTDNDGPSTPYPTLSTADTYPTPPYINVTDET  TDVTDEITDVTTDGPFSSTTPPPEISGGVVVRGDCAFDVNGDLNDPAPIFTPQNQLEWLVPSPSGVVELS  NGAYIDMYCDKSFIAPFSNRTKVTAQCLQKQYFLVDGVIHPFSDFSCTSWPAYTARRTGRPCNGGTDLVE  VGFVLTSGFLQIMDVCHDEVNEVTRYVHHKLNPSSAGYQHGVTRPSFITGDFYAGKNVDNLYTKVQQNNT  ISKILGMDASPFFNDTIDVYLARGHMAAKVDFIFGAPQKATFFFVNAAPQWQMFNGRNWERVEDSVRRYA  SDQALDLDCYTGIWGVSTLPDVNGVQRELYLAFDENNNGLIPVPKLYFRVVIDRKSRNGIVLIGVNNPHV  TLEEIKKDYVICKDVGNRINWVSWDKENLMNGYSYACAVDDFISVVKDLPLDDLYTSGLLGVEALTIENI  PS |
| 9 | >ETN62076.1\|*Anopheles darlingi*-dsRNase1  MARQLVVLLICGAAVLQELFIRGVVSREYIEATTDALEWVLPVSEKKNDGTTDPACSISLHRDLTIMQPL  LLKPGSSQFVWPRLNSTTVELDYGQPLELFCSHGFRDGSPVGKAKSAIVTCEGNDELGYAAGSYNISHFT  CQRPVYHVAERTGGRCYGDSTLIRIGFELAPDRFVQLYEVCFNELQLHSHYVKYNLSPHNGHHQRAVKRP  SFLQGGFYRDLKMNSIYTFVKQHATVQRILGTKARADAVLDSKRGLFLSRGHLAAKSDFIFGSHQRASFW  LMNVAPQWQRFNALNWQRIETAVKEYITANDLWLTVYTGTYGVLELLDGNGDPQQIFLDYDAARDPPGRI  PIPKLFYKVLIDEQTQSGIALIGVNNPHATPEEIAEQYVVCKDVSSAIDWIHWKRDSIPDGYSYACDVNE  FNDVTGHLELVQPIGKLLL |
| 10 | >ETN61460.1\|*Anopheles darlingi*-dsRNase2  MKFAVVSALLLLAAFVGARDIPPRAHQDVLFEEDIPEDVPLAGGYATGCSIRLNGDLPALQPLILVPGTA  NFRYPMTSSGILTLNAGETLELACSNGFELYPEKNSIVAACVIDTQFNYDSKMYTFDQFSCTANWRSVAR  RTERRCYNDATIVEVGFELGARFPKVMEVCHDEVTYHNHYIVHEFTPANAGFQTGVARPGWIQGNFYPGV  NVNTLYTVNMQRETIATILNSQARADELVQTTANGIYMARGHIAARADFVYAPQQNATFWFLNAAPQWQN  FNAGNWERIENSAKSFVASRNINVRVYGGTYGVQTLADANGDHHEIFLDFDPNGRARLQAPKVYYKILHN  EAQNSGIVLIGVNNVHISLEEIRRDYIFCTDVSSRIGWINWDRENLARGYSYACEVNEFNRVTGHLPNLN  VASLLI |
| 11 | >ETN61459.1\|*Anopheles darlingi*-dsRNase3  MKGTLCLLVALLSTSSLVSARDLRHEIPVEVPDVHLGVGFATACSVRMTGDLPRPQPLILRPGTDQFRYP  ATDNGLLQLNAGETLELACQQGFALFPGKNTITISCVLNDQFNYDSQMFAFRDFACTENWLSTARRTAQR  CFNGATIVQIGFELGSRFPRFLDVCHDEVTLDNHYVVHEFTPANAGFQQGVPRPGWYQGDFYPGININGL  YTVNTQRSTLATILNSQARADQLVQGTDNGFFMARGHIAARSDFIYGTQQNGTFWFLNASPQWQTFNAGN  WERIEASVKRFVASRNIHVRVYGGTYGIQTQADGNGDHRQIFLDFNANGRTRVRAPMVYYKILHNEAQNS  GIVLIGVNNIHISLEEIRRDYIFCTDVSSRIGWINWERENLILGYSYACEVNEFNRVTGHLPNLNVASLL  I |
| 12 | >EDS34867.1\|*Culex quinquefasciatus*-dsRNase*  MWNAFGLAALVLLAEIELNFSRIALETSTVDFGDSTGKDYEKDYGCKVSMNKDLNLKQPLFLVPGTKQFI  TPITNTTDLLFHQGEQLELFCTRGFGHTGDKSIVTTCDDANEFVHNGKIYNISQLVCKAPVYHVASRTEE  RCFNNARLVKVGFELDDRFLKLYEVCFDEETLGTHYVKHALYPWNVKHQSSKRPSFIQGDYFPDLKMSKL  YSYDSQRDALARILGSPEHADTFLNKKKDIFLARGHLAARADFVYGSHQRATFWFLNVAPQWQKFNSFNW  QRVETGVKDLIAQRGLEVTVYTGTYGILELPDANGDMQPIFLDFDPNNGGRVPVPKVFYKILHDEWHDAG  IALIGVNNPHATPEQIQQDYVLCEDVSDQIKWLKWKPENILGGYSYACDVNEFNAVTKHLPLGKVEKLLV |
| 13 | >EDS38458.1\|*Culex quinquefasciatus*-dsRNase**  MKTLLCVVLGLAFTIEGRDLPLQEAGSVRFAPTCSLNINNQLPIPQPLILHTGTDQFRYPTSNNRILQLN  AGETVELACDNGFNLAPTKTTIIVSCVFDQTFNYDSTMYQFQEFSCTRNWYSSARDTNEPCNQGATIIEI  GFELGTRFPKIMDVCHNSETFENHWVKHEFRRAHAGYQQGVARPSWYQGEFYPGVNVNTLYTIVRQRQTI  AEILNSQSLADDIVGDTSNGVYMSRGHIAARVDFIYATHQNATFWFLNAAPQWQNFNEGNWLKIEDSTRN  FVAARNIRVTVYGGTYGAHTQTDVNGDQQPIFLDFDRNGVQRLPAPKIYYKILHDEQNKAGIVLIGVNDI  HITSMEQIRNEYMFCEDIGDKVSWINWDRFNLKRGFSYACEVNEFLRKIGHLPELDVPNLLI |
| 14 | >JAV11177.1\|*Nyssomyia neivai*-dsRNase*  HLLPTACEIRISGGGLGEPQPLILNSGATEFIEPKDANGIIRLNPGDDISLFCTTGFQSPSTTNNIIRAT  CTTGTIFIIDGNKGNEMTFSNINCKSYPYHTARKSGKTCGNGAGVDIEVGFIVKERFIELFHICHDDIME  STMYVTHSMTPGNEGYQRSFPRPSWLSSGFFGGKNVDNIYTNVRQNARVAEILGSQELADKYIKPTTTST  YLARGHMAAKVDFIFGSQQRATFWLMNVAPQWQSFNAGNWERVESSTRKMASQRNTHFDVYTGTYGVMTL  PDINGQHQEIYLYFDENNNGQIPVPKIYYRVLYEKSTKRGIVLIGVNNIHITAEEAEEQNYIICEDVSDK  INWINWERNDQILGYSYACEVEEFTKVVEHFPKLEISGLYV |
| 15 | >JAV11176.1\|*Nyssomyia neivai*-dsRNase**  IYYLYSQISFSDTSISDECQLCWHSIHSQVKNVIKTSSGTSASQSYYVREVWSIMRVVLFVSVLLHWQLS  SARIPINDHHVISPLMEPCEIRISGGGLGEPQPLILNSGATEFIEPKDANGIIRLNPGDDISLFCTTGFQ  SPSTTNDIIRATCTTGTIFIIDGNKGNEMTFSNIFCKSYPYHTARKSGKTCGNGAGVDIEVGFIVKERFI  ELFHICHDDIMESTMYVTHSMSPGNEGYQRSFPRPSWLSSGFFGGKNVDNIYTNVRQNARVAEILGSQEL  ADKYIKPTTTSTYLARGHMAAKVDFIFGSQQRATFWLMNVAPQWQSFNVGNWERVESSTRKMASQRNTHF  DVYTGTYGVMTLPDINGQHQEIYLYFDENNNGQIPVPKIYYRVLYEKSTKRGIVLIGVNNIHITAEEAEE  QNYIICEDVSDKINWINWERNDQILGYSYACEVEEFTKVVEHFPKLEISGLYV |
| 16 | >AAF49206.1\|*Drosophila melanogaster*-dsRNase1  MKCLVLIAFSGLFLASAQARILPAEDLPWELPEVPVAVNEIEPRAAGCSIKIRSSELKDPQPLLIKSDTS  EIVGFSDTGYVDVDKDKTIEFHCTSSLASPLSGKSVTAKCVGGTTFKIDDKEHDLSAIKCTSWPVFVGKK  SGSSCNGGTTLIKVGFELSGSRFATQYEVCFNEDEEVTRYVYHRLEPGNNYYATGVDRITFGAGGYFAGK  NVDKLYTQAVQKETIDKELDMDSSRFFDSAKNIFLARGHMGAKADFVFAPEQRATFLFINAAPQWQTFNA  GNWARVEDGVRAWVAKENKHVECWTGVWGVTTLPNKNGEQRQLYLSHDNNGNGLIPVPKLYFRVVIEPST  KKGIVLIGVNNPHLSLEEIKRDYILCTDVSDRINWISWKKTDITAGYSYACEVPEFRKKVTHLPEFSVSG  LLV |
| 17 | >AAF49208.1\|*Drosophila melanogaster*-dsRNase2  MKCIRFSLLVVGLLAAPAAWARVPCPEVELPPVVEDDGIFERIAVAPPQPVGRAGACSVTIRGGLPSPEP  VYLKTDSEDFYPFSDVGVMEFESGGSLQLWCPSGFNTHSENLLTASCVSGTTFSVGGSNFEFKDLYCKSW  PGFKAVKSGATCNGGIVIRVGFEITSSRFAEQMQICFNEEEEVTRYTRHKLEPGSNYYETGVARITFQTA  GFFDGKNVDKLYTQATQLETINNELGGDAEKYFDSSSNVYLARGHLGAKADFDYAPEQRATFLFINAAPQ  WQTFNAGNWARVEDGLRAWVSKNKLNVNCYTGVYGVTTLPNKDGVETPLYLAKDDNNNGLIPVPKLYFRV  VIDPSSHRGIVFVGVNNPHLTEEQIKRDYVICDDVSDQVTYINWKTTDIKAGWSYACEVADFLKTVKHLP  ALTAKGGLLV |
| 18 | >XP_320813.4\|*Anopheles gambiae*-dsRNase  SECTVNIRTQLNAREPLFLRNNQLWAPNGPSLQWNAGETTLIACPGNTIQNTGTVTANIQCVSGTTFNLA  GSNVNIADVSCTARSTGSHQTTGQSCGSGGTLLNLGFDVPGVGFVTYIQSCYNMQTASVIYTRHIIPGAA  ISHSISESYRPSFKTAGTAPHVQPATSYTTAQQAIRFAQLLGSQAQADRFITSSSYLSRGHLSPDADGIF  RPWQWATYFYVNVAPQWQATNGGNWLVVENAARNIAGRLNEDVLIFNGAHDILTLPHVNGQQVPITLEAG  GIQTPKWYWKIIKSPRTNAAIALVNNNDPFRTSMPAGEMLCQDVCGQYGWGNANYGNFARGFTYCCTVAD  LRRAIPSIPAEADAANVLRF |
| 19 | >XP_018803412.1\|*Bactrocera latifrons*-dsRNase1  MKLTSTLLLLVAGSFFLFHGCTAGVVAVPEDVVDDWKAEDHTSELINILFERNLYVKRIKEDASQAIVPV  EAEEAEEDYVEPEPISEVEDELVQPEVDVNGKIEGRASECKVTIRGGLPTPQPLYLKSGSEEIYPYDTKG  VMVVNAGRTLEMWCPGKFTKLDTTLVTATCVSGTNFRVDGTTYAFKELTCKAWPTFVAEKTGASCNGGVM  VRVGFKISSTRFAQQYEVCFNEGEEVTRYVHHDLNPGANYYQTGVDRITFQTGGFFDGKNVDKLYTQATQ  LETINAHLGGDARKYFDSAKNVYLARGHMAAKADFDYGLEQRATFLFINAAPQWQVFNAGNWARIEDGVR  AKVSSARWYVDCYTGVYGVTTLPNSDGVQTPLYLAYDSNNNGLIPVPKLYFRVVIEKTSKKGIVFIGVNN  PHLTLDEIKKDYIICTDIADRVKYINWKRTDVTAGYSYACEVSDFRSKVTNLPNLSAPGGLLL |
| 20 | >XP_018803418.1\|*Bactrocera latifrons*-dsRNase2  MYSGGKAVKLALLLACLLADVEAGILHSKETAVKSLLPNTENDGPRTPYPTLSTADTHPTPPYINVTDET  TDVTTDGPFSSTTPPPEISGGVVVRGDCAFDVNGDLNDPAPIFTPPNQLEWLVPNPAGVVELSNGAYIDM  YCDTSFIAPFSNLTKVTAQCLQKQYFLVDGVIHPFSDFSCTSWPAYTARRTGRPCNGGTDLVEVGFVLAS  GFLQIMDVCHDEVNEVTRYVHHKLNPSSAGYQHGVTRPSFITGDFYAGKNVNNLYTKVQQNNTISKILGM  DASPFFNDTIDVYLARGHMAAKVDFIFGAPQKATFLFVNAAPQWQMFNGRNWERVEDSVRRYASDQALDL  DCYTGIWGVSTLPDVNGVQRELYLAFDENNNGLIPVPKIYFRVVIDRKSRNGIVLIGVNNPHVTLEEIKK  DYVICKDVGKRIKWVSWDKENLMNGYSYACAVDDFISVVKDLPLEDLYTSGLLGVEELTIENIPS |
| 21 | >XP_014088323.1\|*Bactrocera oleae*-dsRNase1  MKLASTLLLLVAGSFCLFHGCIASVIATPQHVNDESKADDQLSEPIAPISAINEEDKLEGLETLFERNLY  VEPIKGADYQYIKSAAVSVDEKPAEVEEDYVEPEPNSEVEDELVQPEADVNGKPEGRANECKVTIRGGLP  TPQPIYLKSGSEEFYPYDTRGVMVVDSGKTLEMWCPGKFTTIDKTLLTASCVSGTNFRVDGTTYSVKELT  CKSWPGFVAEKTGASCNGGIMVRVGFKVSSRRFVEQYQVCFNEDEEVTRYVHHDLNPGSNYYQTGVDRIT  FQTGGFFDSKNVDKLYTQVTQQATINAHLGGDASKYFTSNKNIYLARGHMAAKADFDYGSEQRATFLFIN  VAPQWQVFNAGNWARIEDGVRAKVSSAKWYVDCYTGVYGVTTLPNSDGVQTPLYLSYDSNNNGQIPIPKL  YFRVIIERSSQKGIVFIGVNNPHLTLNEIKKDYIICNDISDRVNYVNWKRTDITAGYSYACEVSDFRRKV  THLPTLSAPGGLLL |
| 22 | >XP_014088332.1\|*Bactrocera oleae*-dsRNase2  MYSGGKAVQLTLSLVCLLAAVEAGILYSKEPAVKSLILNTKDDGPRTPYRTSSTAATYSTPSFINVTDGP  TDVTTDGPFSSTTPPPEISAGVVVRGDCAFDLNGDLNDPAPIFTPHNQLEWLVPNPAGIVELSNGAYIDM  YCNKSFIAPFSNSTKVTAQCLQNQYFLVDGLIYPFSNFSCTNWPAYTARRTGRPCNGGTDLLEVGFELPT  GFLQIMDICHDEVNEVTRYVHHNLNPSSAGYQHGVSRPSFITGDFYAGKNVNNLYTKVQQNRTISRILGM  DASRFFNDTLDVYLARGHMAAKVDFIFGASQKATFYFVNAAPQWQMFNGRNWERVEDSVRRYASDKALDL  DCYTGIWGVSTLPDVNGIQRELYLAFDENNNGLIPVPKIYFRVVIDRKTRNGIVLIGVNNPHVTLEEIKK  DYVICKDVGNRIKWVSWDKENLMNGYSYACAVDDFISVVKDLPLDELYTSGLLGVEELIIENIPL |
| 23 | >XP_017466898.1*\|Rhagoletis zephyria*-dsRNase1  MVVDTGSSLQMWCPGQFSSIADTLITATCVSGSNFRVSGTTYAFKELACKAWPAFVAEKTGASCSGGIMV  RVGYKISSSRFAQKYEVCFNEEEEVTRYVHHDLDPGSNYYETGVARITFQTAGFFDGKQVDNLYKQVTQQ  ATIEAQLGNDASQYFDSNKNVYLARGHMAAKADFDYASGQRASFLFINAAPQWQVFNAGNWERIEDGVRS  KVAASKWYVDCYTGVYGVTTLPNADGVQTPLYLAYDSNNNGLIPVPKLYFRIVIERTSQKGIVFIGVNNP  HLSLEEIQKDYILCADVADQVDYINWKRTDLTAGYSYACTVDDFKQNVTYLPEVSASGGLLL |
| 24 | >XP_017478492.1\|*Rhagoletis zephyria*-dsRNase2  MANTKRTLELFFALLCFSVAVQAGLLQSKQRQRYTELSTVVPAVEGTDPTPPSNITTDSP  NNSTTDSPFNTTLTAPPSTATSPPDSGSGVVVRGDCAFDINGELNDPAPIFTPRNHFDWL  VPNAAGVVELSNGAYIDMYCSTSFMAPFTNRTKVTAQCLQKKYFLVDGLIYPFANFSCTA  WPAYTARRTGRPCNGGTDLLEVGFELEAGFLPTMDICHDEVNEVTRYVHHVLNPSSNGYQ  HGVSRPSFITGDFYNGKNVNNLYTKVEQNKTISQILGMDASPYFNDTIDVYMARGHMAAK  VDFIFGAPQKATFYFVNAAPQWQMFNGGNWERIEDGVRRFASDQALTLDCYTGIWGVSTL  PDVNGVQQELYLAFDENNNGLIPVPMLYFRVVIDRESRKGIVLLGVNNPHISLEEIKRDY  VICKDVGRRIDWIGWNKENLMKGYSYACAVDDFLKVVKHLPLEDLYTTGLLGVEELKIEN  APIWEEH |
| 25 | >XP_004530585.1\|*Ceratitis capitata*-dsRNase1  MKLTTTLLLLVTGSCCLLQGCTAGVIAVPEVVIDGLKIDEEKPEESVFERNLYVEPISGAEDDYVEPEDE  LLPPAPVVDDKPKGRATACKVTIRGGLPTPQPVYLKSDSAEFYPYDSTGVMVVESGEKLDLWCPGKFTSL  DKTLVSASCVSGTNFKVDGTTYTLKELTCKSWPSFVAEKTGSSCNGGVEVRVGFKVSSSRFVEQYKVCFD  EDEEVTRYVHHDLNPGSNYYQTGVDRLTFQTGGFFDGKNVDKLYTQATQLTTINEQLGGDASKYFDSSKN  VYLARGHLAAKADFDYGTEQRATFLFINAAPQWQVFNAGNWARIEDGVRAKVSKAQWYVDCWTGVYGVTT  LANANGVQTPLYLAYDSNNNGLIPVPKLYFRVVIERTTKKGIVFIGVNNPHLTLAEIKKDYILCTDVADK  VDYVNWKPTDITAGYSYACEVDDFKKKVSHLPDLPSVTGLLV |
| 26 | >XP_005177226.1\|*Musca domestica*-dsRNase1  MKLATSLLLLVAGFACLIHVSVGGVVGVPTDLLENLKIADDLPEAPPVVEEEIFERNVVVDVLPEGRANE  CQITIRTGLSEPQPVFLKTNAAEFYPYSNTGVMQVHAGGTLQMFCPGEFKVKATKLITATCVSGTTFKVD  GTSYAFSELVCKSWPGFVAKKKGTTCNGGILVGVGFEVSSTRFVEQMEICYNEQEEVTRYVRHTLGPASN  YYQTGVDRITFQTAGFFNGKNVDKLYTQATQLETINAELGGNAGKYFDSSKNIYLARGHMGAKADFMYGT  QQRATFLFINAAPQWQVFNAGNWARVEDGVRAWVSKNSKTVNCYTGVYGVTTLPNKNGVQTPLYLAHDSN  NNGLIPVPKLYFRVVIEPATKKGIVFVGVNNPHLTLEQIKKDYIICTDVSSKVNYISWKKDDITAGYSYA  CEVADFLKTVKHLPALTATGGLLV |


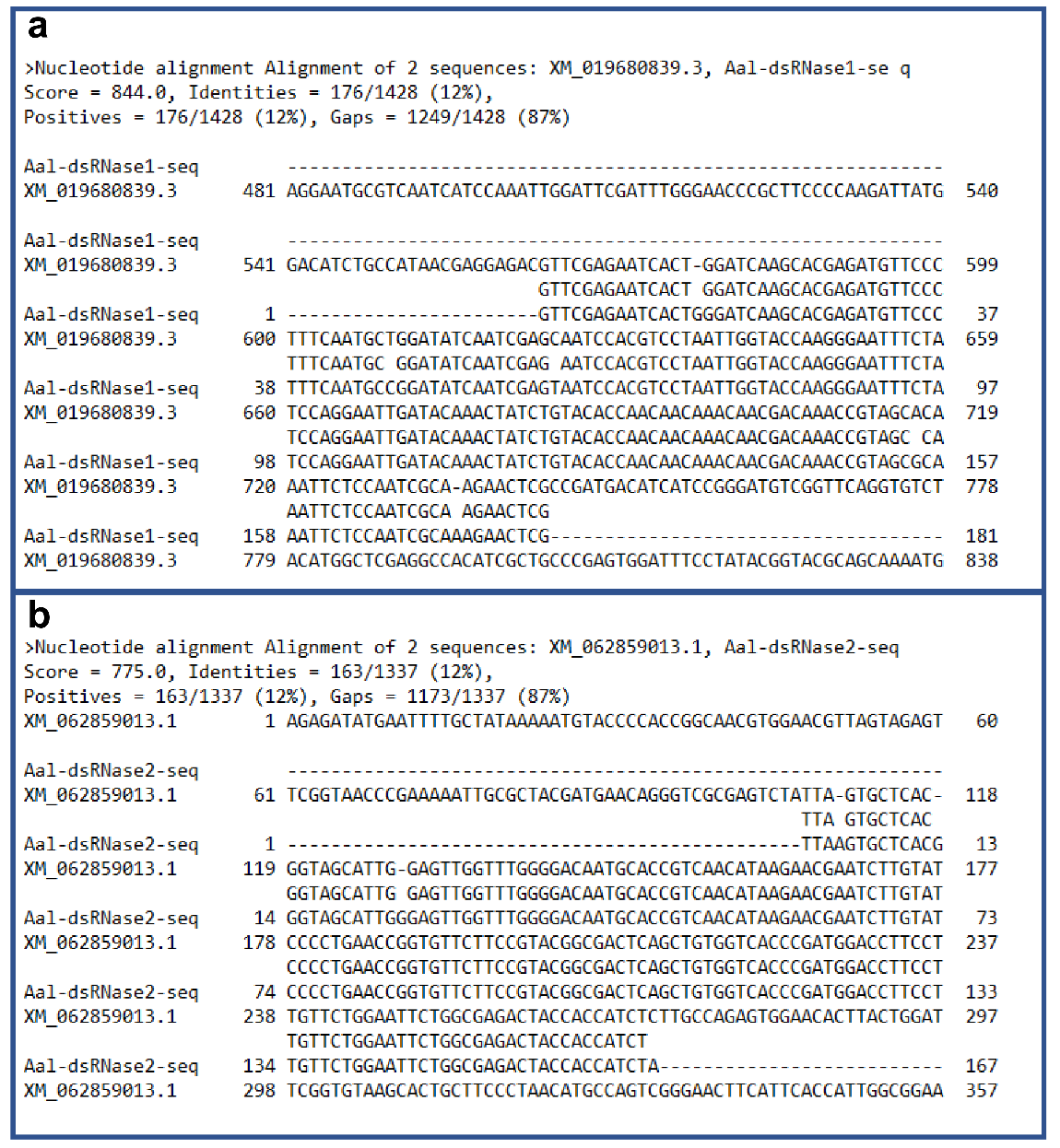


**Fig. S5**. Sequencing data of dsRNase genes of *Aedes albopictus*. The RT-PCR products of the (**a**) Aal-dsRNase1 and (**b**) Aal-dsRNase2 were sequenced and aligned with the corresponding reference sequences using Geneious Prime v2025.1.

**7 Protection of dsRNA from degradation by *Aedes albopictus* gut extract using commercially available transfection reagents**


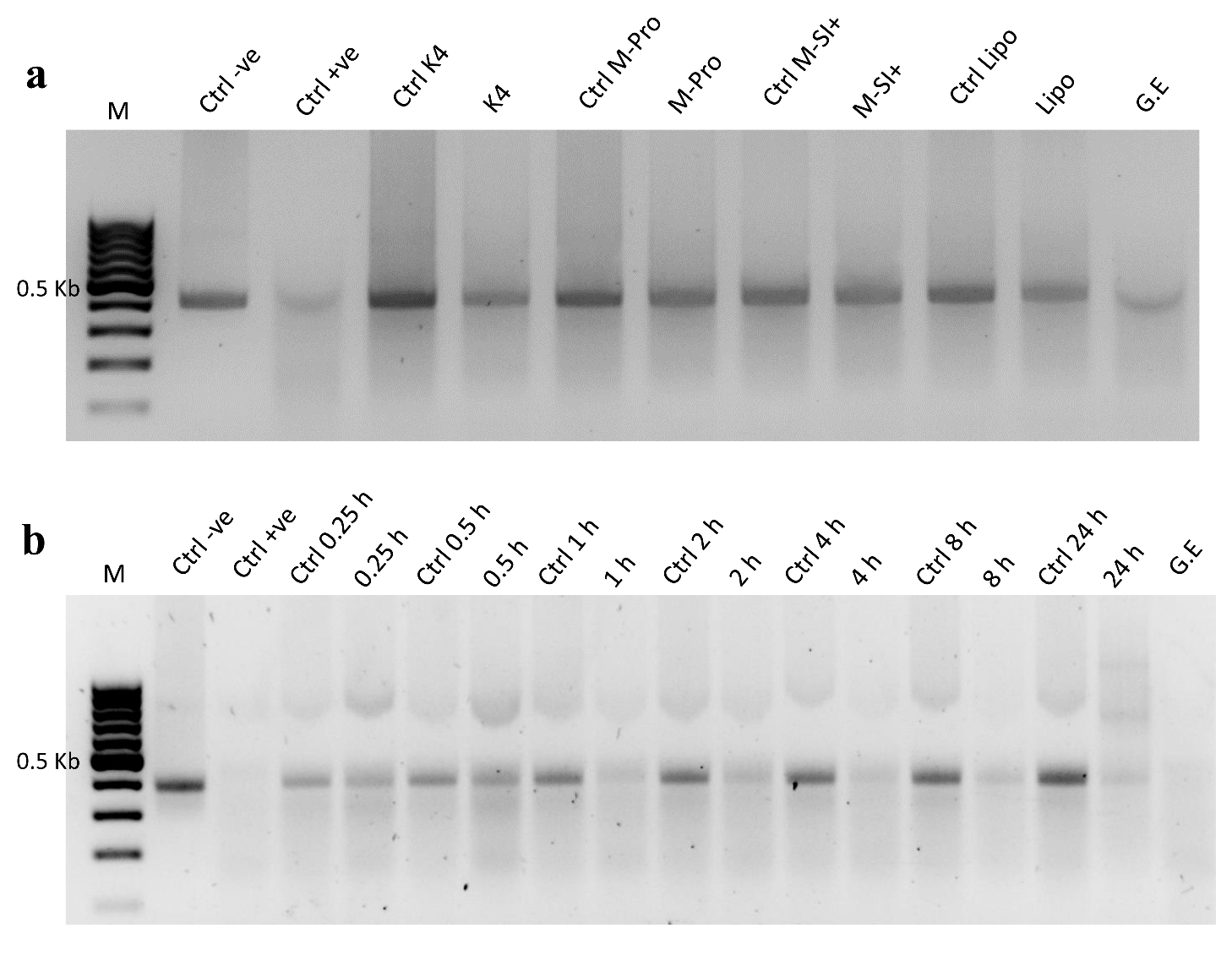


**Fig. S6**. Protection of dsRNA from degradation by *Aedes albopictus* gut extract. The dsRNA was complexed prior to the degradation assay in (**a**) with K4, Metafectene-Pro (M-Pro), Metafectene-SI+ (M-SI+), or Lipofectamine-2000 (Lipo). In (**b**), dsRNA was complexed with K4 and incubated with gut extract for 0.25 to 24 h. EDTA was added post-incubation to stop degradation, and samples were analyzed by agarose gel electrophoresis. Negative control (Ctrl -ve) was incubated in nuclease-free water while positive control (Ctrl +ve) was incubated in gut extract. Gut extract containing EDTA served as the independent control for each dsRNA:TR sample and time point. A GeneRuler 100 bp DNA ladder (M, Thermo Fisher scientific) was used as marker. The image shown are representative of three independent experiments (n=3). G.E = gut extract alone.
